# Double Burden of Malnutrition Prevalence in Pregnant Women: Systematic Review and Meta‐Analysis

**DOI:** 10.1111/jhn.70347

**Published:** 2026-09-17

**Authors:** Amanda Biete, Camila Biete, Vivian S. S. Gonçalves, Erika S. O. Patriota, Nathalia Pizato

**Affiliations:** ^1^ Graduate Program in Human Nutrition University of Brasilia, Campus Universitário Darcy Ribeiro Brasília DF Brazil; ^2^ Graduate Program in Public Health University of Brasilia, Campus Universitário Darcy Ribeiro Brasília DF Brazil

**Keywords:** double burden of malnutrition, gestational diabetes mellitus, nutritional deficiency, obesity, pregnancy, underweight, vitamin D deficiency

## Abstract

**Introduction:**

The coexistence of undernutrition and overweight, obesity, or diet‐related noncommunicable diseases and micronutrient deficiency in pregnant women is defined by the WHO as a double burden of malnutrition (DBM). These coexistences are a critical aspect of maternal and antenatal care, especially in low‐ and middle‐income countries.

**Objective:**

To estimate the global prevalence of DBM in pregnant women through a systematic review and meta‐analysis.

**Methods:**

The systematic review was conducted in accordance with the PRISMA recommendations. Studies that reported prevalence measures of DBM in healthy pregnant women were included. DBM was considered as the coexistence of underweight with gestational diabetes mellitus (GDM), underweight with hypertension, overweight/obesity with anemia, overweight/obesity with vitamin D deficiency, overweight/obesity with vitamin B12 deficiency, and overweight/obesity with folate deficiency. The meta‐analysis used a random‐effects model with inverse‐variance weighting. Risk of bias was assessed using Joanna Briggs Institute tools, and the certainty of the evidence was evaluated using the Grading of Recommendations Assessment, Development, and Evaluation (GRADE) framework.

**Results:**

The systematic review included 62 articles totaling 1,386,245 pregnant women, at 8–42 gestational weeks and 13–45 years of age. The most common DBMs identified were overweight/obesity and vitamin D deficiency (21%; 95% CI: 14%–27%, *n* = 10 studies) and overweight/obesity and anemia (9%; 95% CI: 7%–10%, *n* = 18 studies). A meta‐analysis of overweight/obesity and micronutrient deficiency was not conducted due to the small number of eligible studies. Most studies (66.3%) had high methodological quality; however, GRADE rated the overall certainty of evidence as low.

**Conclusion:**

A high prevalence of overweight/obesity with vitamin D deficiency and anemia were observed. These results emphasize the need for stronger public policies to ensure equitable access to healthy diets, evidence‐based supplementation, and continuous nutritional monitoring during pregnancy.

## Key Points

- A systematic review and meta‐analysis estimated the global prevalence of DBM in pregnant women.
- The most common DBM combination was overweight/obesity and vitamin D (21%) deficiency and overweight/obesity and anemia, with a pooled prevalence of 9%.
- Most studies (66.3%) had high methodological quality.
- Findings reinforce the importance of strengthening public policies that ensure access to healthy foods, adequate supplementation, and nutritional monitoring during pregnancy.

## 1 Introduction

The World Health Organization (WHO) defines the double burden of malnutrition as the coexistence of both undernutrition (such as stunting, wasting, and micronutrient deficiencies) and overnutrition (such as overweight and obesity) in the same population, community, household, or even individual [1]. Also, the WHO describes the coexistence of malnutrition and resulting diet‐related noncommunicable diseases (NCDs) such as heart disease, stroke, diabetes, and some cancers, leading to serious long‐term health and social consequences [1, 2], especially in low‐ and middle‐income countries (LMICs) [3].

Globally the high rates of undernutrition and micronutrient deficiencies alongside overweight/obesity at the individual, household, and population levels are reported [4]. This phenomenon highlights the complex and multifaceted nature of DBM combination. Besides, this is related to a series of global changes, including nutritional, demographic, and transitions/overlaps in epidemiological factors. These transitions encompass shifts in dietary patterns, population dynamics, and disease profiles that have occurred worldwide [5, 6]. Likewise, the overlap occurs because many LMICs are still dealing with the unfinished agenda of undernutrition and infectious diseases, while simultaneously experiencing a rapid rise in obesity and NCDs. This places an immense strain on their healthcare systems, which must address both types of burdens simultaneously [3].

The first thousand days of life, from conception to 2 years of age, are considered a critical period for human growth and development [7]. Maternal malnutrition and micronutrient deficiencies, such as iron, folate, and vitamin D, have also been prevalent in pregnant women, which can lead to several adverse outcomes for both mother and child [8, 9]. Therefore, focusing on adequate nutritional status and access to healthy foods is essential to ensure maternal‐child health during pregnancy [10].

Most studies on DBM coexistence have focused on the population and household levels [11], while the individual level remains understudied. Globally, DBM prevalence at the household level is generally below 10%, but in LMICs it is increasing, particularly in Latin America [11]. At the individual level, the most prevalent manifestation DBM is the coexistence of overweight/obesity and anemia [12], but the data point to particularly high rates among adult women (aged 20–49) when compared to adolescent girls (aged 15–19), reaching prevalence estimates as high as 30% in LMICs such as Togo and Ghana [13]. Despite its increasing prevalence, individual DBM in pregnancy is insufficiently studied to define causes and associated factors, particularly concerning women of reproductive age, thereby hindering progress toward global nutrition goals [14].

Malnutrition related to micronutrient deficiencies and diet‐related NCDs at the individual or family level are poorly investigated as components of DBM combination. Also, studies evaluating the coexistence of micronutrient deficiencies and NCDs, such as obesity, at the individual level during pregnancy are scarce. Based on this background, this study aims to investigate the prevalence of individual DBM in pregnant individuals through a systematic review and meta‐analysis. The findings of this study may provide valuable evidence and insights into new findings, which policymakers and organizations can utilize to develop more assertive targeted prevention strategies for addressing the DBM during pregnancy.

## 2 Methods

### 2.1 Registration and Protocol

This systematic review was registered in the International Prospective Register of Systematic Reviews (PROSPERO), number CRD42024536936, and was conducted according to the Preferred Reporting Items for Systematic Reviews and Meta‐Analyses PRISMA checklist [15] (Appendix S1).

### 2.2 Eligibility Criteria for the Studies

Observational studies that presented prevalence data on individual DBM combination in pregnant women, or presented data about body mass index, gestational weight gain, micronutrient deficiency, anemia, hypertension, and gestational diabetics were also considered for this review. No restrictions by country, language, or publication date, were eligible for this systematic review.

The following criteria were excluded from the review: studies conducted with pregnant women with communicable diseases or surgeries prior to pregnancy; studies conducted only with adolescents; reviews; qualitative studies; abstracts; book chapters; and publications without primary data.

### 2.3 Search Strategy

The search was carried out on June 19, 2025, in the electronic databases Medline, Embase, Scopus, Web of Science, Lilacs (BVS), and SciELO (searches in English and Spanish), and in the gray literature in the Google Scholar and ProQuest Dissertations and Theses Global databases. The reference lists of the studies included in the review were manually searched for additional relevant publications. The Google Scholar search was limited to the top 200 most relevant articles [16]. Data not found in the evaluated articles were requested by email to the authors. No language, publication date or status filters were applied to the searches. The search strategies in each database were detailed in Appendix S3.

The search strategy was conducted according to the criteria established by the Peer Review of Electronic Search Strategies (PRESS) checklist [17]. The keywords used in the search strategy were found in Medical Subject Headings and Health Sciences Descriptors. Researchers with experience in conducting systematic reviews reviewed the search strategy, following the PRESS checklist [17].

The search strategy was adapted for each database using the following Boolean descriptors and operators: (Pregnancy OR “Pregnant women” OR Gravidity OR Pregnant OR Gravid OR Gestation) AND (anemia OR anemia OR “iron deficiency anemia” OR “iron deficiency anemia” OR “Iron deficiency” OR “Iron‐Deficiency Anemia” OR “Maternal anemia” OR “Deficiency Diseases” OR Avitaminosis OR “Nutritional Deficiencies” OR “Vitamin Deficiencies” OR Hypertension OR Diabetes OR Hyperglycemia) AND (“Double burden of malnutrition” OR “Nutritional Deficiency” OR “Nutritional Deficiencies” OR “maternal obesity” OR “Obesity in Pregnancy” OR “gestational weight gain” OR “maternal overweight” OR Undernutrition OR Underweight OR Undernourished OR Stunting OR Wasting) AND (“Observational Study” OR “Cohort study” OR “Longitudinal study” OR “Follow‐up study” OR Cohort OR Longitudinal OR Prospective OR Retrospective OR “Incidence study” OR Follow‐up OR “Prevalence study” OR Prevalence OR “Cross‐Sectional Study” OR Cross‐Sectional OR frequency).

### 2.4 Studies Selection

Two reviewers independently conducted the review selection process in two steps. First, the titles and abstracts of the studies were read, selecting those that met the eligibility criteria. The records found in the databases were downloaded into a Microsoft Excel spreadsheet and imported into Mendeley to merge them into a single file and remove duplicates. Subsequently, the Rayyan software was used to identify additional potential duplicate references and screen potentially eligible studies. Subsequently, the pre‐selected studies were read in full to confirm the selection and then perform data extraction. Any disagreements were resolved by consensus.

Some studies did not provide the information necessary for definitive inclusion in the review, so at least two attempts were made to contact the authors to obtain additional information or to request the full text when it was not available in the article.

### 2.5 Data Extraction

Study characteristics were extracted independently by two reviewers using a standardized data extraction form. The following information was extracted: author's name, year of study, title, year of publication, country, study design, local data collection, age of pregnant women, gestational trimester, sample size, anemia, hypertension, gestational diabetes mellitus, vitamin deficiency, collection method, and prevalence of double burden of nutrition. The Google NotebookLM tool was used to support the organization, and verification of information extracted from the included studies, used only for preliminary synthesis and support, without replacing the critical evaluation performed by the authors. Attempts were made to contact the authors to obtain additional information when this data was not available in the articles or when the full text could not be accessed. These data were used to summarize the characteristics of the included studies and to investigate potential sources of heterogeneity through predefined subgroup analyses and meta‐regression.

For articles that did not provide a 95% CI for the prevalence of double burden of malnutrition, it was calculated retrospectively using the website OpenEpi [18] with the sample size and the number of events.

### 2.6 Assessment of Methodological Quality

The two investigator authors assessed the methodological quality of each included study using the JBI Critical Appraisal Checklists for Cohort Studies, Analytical Cross‐Sectional Studies, and Case‐Control Studies by the Joanna Briggs Institute [19]; however, these checklists were not used as a criterion for exclusion. The risk of bias was considered low when the answer to all items was “yes” and high if the answer was “no” or “unclear” to at least one of the items.

The instrument for Cohort Studies consists of 11 questions from four perspectives (yes, no, unclear, and not applicable) which consider criteria such as similarity between groups, validity and reliability in measuring exposure and outcomes, control of confounding factors, adequacy of follow‐up time and statistical analyses, and management of losses during follow‐up [20].

The instrument for Analytical Cross‐Sectional Studies consists of eight questions that address criteria such as clarity of inclusion criteria, description of participants and setting, validity of exposure and outcome measures, identification and management of confounding factors, and appropriate statistical analysis [20].

The instrument for Case‐Control Studies consists of eight questions which assess comparability between cases and controls, the adequacy of selection and matching criteria, the validity and standardization of exposure and outcome measurements, and the identification and control of confounding factors. Relevant exposure time and appropriate statistical analysis are also considered [20].

Any disagreements were resolved by consensus. An analysis of the relative frequency of each investigated domain was presented, and no scores were assigned.

### 2.7 Summary Measures and Synthesis of Results

The primary outcome of the analysis was the prevalence with 95% confidence interval (95% CI) of double burden of malnutrition.

The meta‐analysis used a random‐effects model estimated by the DerSimonian–Laird method. The meta‐analyses were performed whenever at least five independent prevalence estimates were available for the same outcome and the studies were considered sufficiently homogeneous from a clinical and methodological perspective to justify statistical pooling.

The prevalence and confidence intervals of each study were presented in a forest plot. Statistical heterogeneity between studies was measured using I‐squared (*I* ^2^) [19]. Heterogeneity was not considered important when *I* ^2^ values were less than 40% [21].

### 2.8 Assessment of Heterogeneity and Publication Bias

Subgroup analyses were performed to identify the source of heterogeneity among the studies included in the systematic review. The subgroup analyses were undertaken whenever at least two independent prevalence estimates were available within each subgroup and the available data allowed meaningful interpretation. In the subgroup analysis, the following covariates were used: Continent (Africa, Asia, Europe, North America, and South America), gestational trimester (all, only in one or two trimesters, and not reported), methodological quality (high or low), and life cycle (adult, adults and adolescent, and not reported (NR)).

Small‐study effects were explored using funnel plots and Egger's regression test, and in meta‐analyses of prevalence, funnel plot asymmetry may reflect between‐study heterogeneity, methodological differences, or the bounded distribution of proportions rather than publication bias alone. Publication bias was assessed using the Egger test at the 5% significance level and by visual inspection of the funnel plot [22]. Data analysis was performed using Stata version 17.

### 2.9 Certainty of the Evidence

The overall quality of the evidence was summarized using the GRADE system. Following the GRADE approach for prevalence studies, the certainty of evidence started at high and was downgraded, when appropriate, based on risk of bias, inconsistency, indirectness, imprecision, and publication bias. The GRADE assessment was conducted independently by two reviewers, as recommended by the GRADE Handbook criteria [23] and disagreements were resolved by consensus between the reviewers. The rating could be downgraded or upgraded based on the following criteria [1]: Risk of bias (assessed with the Joanna Briggs Institute tool, including whether more than 75% of studies had inadequate sampling, inappropriate statistical analyses, or significant loss to follow‐up) [2]; Inconsistency (heterogeneity considered notable when *I* ^2^ > 40%) [3]; Indirectness (downgraded if more than 75% of studies did not use valid and reliable data collection methods) [4]; Imprecision (downgraded if more than 75% of studies had small samples, ≤ 500); and [5] Publication bias (considered present when *p* < 0.05).

## 3 Results

### 3.1 Selected Studies

**FIGURE 1:**
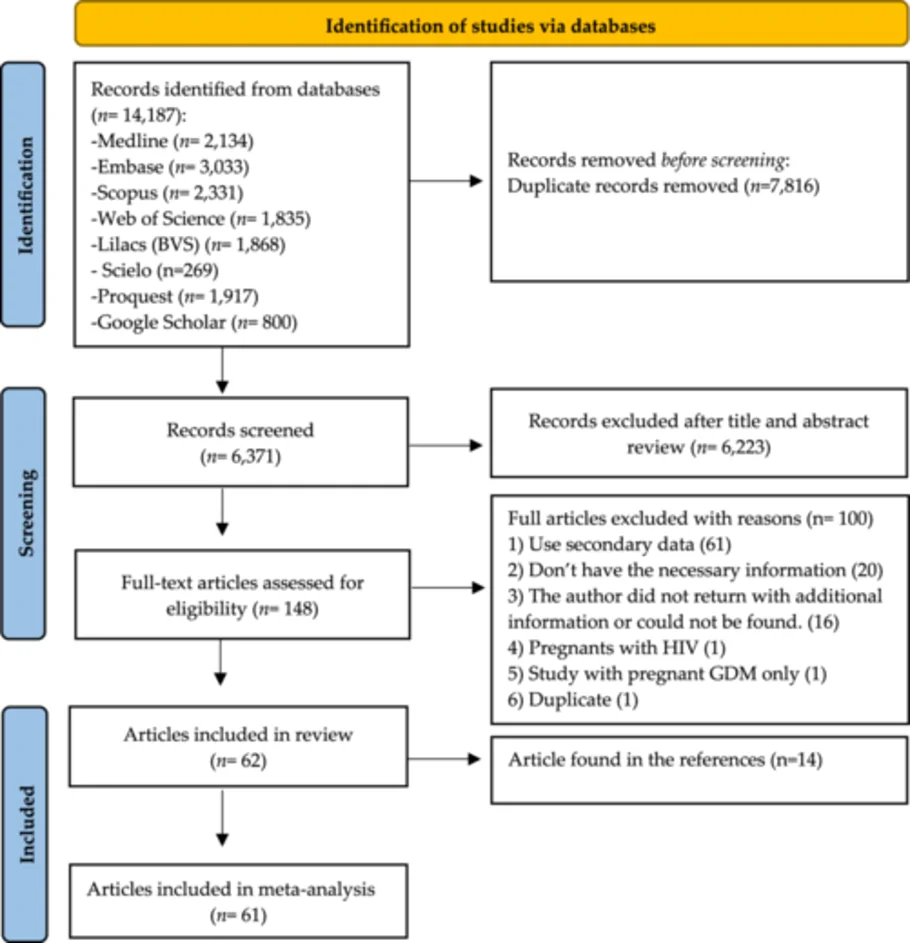
Flow chart of the literature search, selection criteria, and inclusion of studies (adapted from PRISMA).

The search included eight electronic databases and gray literature, identifying a total of 14,187 records. After removing duplicates, 6371 were screened based on titles and abstracts. Finally, 148 full texts with potential for inclusion were pre‐selected for reading. Of these, 100 were excluded because they did not meet the eligibility criteria (Figure 1). The articles excluded from the review are available in Appendix S4. After full reading, 49 studies were included, and 14 were added from the reference lists of other studies, totaling 62 articles included in the systematic review.

### 3.2 Characteristics of the Studies

Considering the 62 selected articles, 1,386,245 pregnant women from all gestational trimesters, ranging from 8 [24] to 42 [25, 26, 27] weeks and ages 13 [28, 29] to 45 [30, 31, 32, 33, 34], were included in the analysis. Regarding study design, 45 (72%) [24, 26, 28, 30, 31, 32, 33, 35, 36, 37, 38, 39, 40, 41, 42, 43, 44, 45, 46, 47, 48, 49, 50, 51, 52, 53, 54, 55, 56, 57, 58, 59, 60, 61, 62, 63, 64, 65, 66, 67, 68, 69, 70, 71, 72] were cohort studies, 16 (25%) cross‐sectional [25, 27, 29, 34, 73, 74, 75, 76, 77, 78, 79, 80, 81, 82, 83, 84], and one (1,6%) was a case‐control study [85]. Among the selected articles, 33 (53%) were conducted only with adult pregnant women [24, 25, 30, 31, 32, 33, 36, 37, 40, 41, 46, 47, 50, 51, 54, 56, 57, 58, 60, 62, 64, 65, 66, 68, 71, 73, 74, 76, 77, 78, 82, 84, 85], and 21 (33%) also included adolescents in the sample (according to each author's criteria) [27, 28, 29, 34, 35, 38, 42, 44, 48, 55, 59, 61, 67, 69, 70, 72, 75, 79, 80, 81, 83]. The articles were published from 1998 to 2025. Thirty‐nine studies (62%) were conducted in Asia [24, 26, 29, 31, 33, 35, 36, 38, 41, 42, 43, 44, 45, 46, 47, 48, 49, 50, 51, 52, 53, 54, 55, 56, 57, 58, 59, 61, 62, 63, 64, 65, 66, 68, 74, 75, 76, 81, 82], 12 (20%) in Europe [30, 32, 37, 39, 40, 60, 67, 69, 70, 71, 79, 85], six (10%) in South America [25, 27, 78, 80, 83, 84], three (5%) in Africa [34, 73, 77], and two (3%) in North America [28, 72].

Of the 62 articles, most were conducted in urban or mixed populations, while only two were conducted solely in rural populations [28, 75]. Regarding the gestational trimester, only 23 (37%) articles evaluated pregnant women across all gestational trimesters [33, 40, 41, 44, 45, 46, 51, 52, 54, 55, 56, 57, 59, 61, 64, 66, 71, 76, 78, 79, 81, 83, 84].

The profiles of the pregnant women evaluated in the accompanying articles are quite diverse in terms of educational attainment, income, and race/ethnicity, reflecting the different geographic and socioeconomic contexts of the studies. Regarding education, the pregnant women range from lower educational degrees to graduate degrees. When income and socioeconomic status were evaluated, some studies identified a significant rate of pregnant women facing more disadvantaged socioeconomic conditions. Lastly, regarding race and ethnicity, many studies have focused on specific populations, with a strong representation of Asian pregnant women, especially Chinese.

Detailed information on the definition criteria for each coexistence evaluated as DBM, such as undernutrition, obesity, anemia, GDM, hypertension, and deficiencies in vitamins D, B12, and folate, is provided in Appendix S2. Mostly studies evaluated the nutritional status by using pre‐gestational body mass index (BMI); GDM Diagnostic Criteria by International Association of Diabetes and Pregnancy Study Group (IADPSG); Hypertension Diagnostic Criteria by systolic blood pressure ≥ 140 mmHg or diastolic blood pressure ≥ 90 mmHg after 20 weeks of gestation; Anemia Diagnostic Criteria by WHO criteria of hemoglobin serum levels; Vitamin D deficiency diagnostic criteria under < 20 ng/mL; Vitamin B12 deficiency (< 150 or 200 pg/mL); and Folate deficiency (< 3 or 7 ng/mL).

### 3.3 Results of Individual Studies

Mostly of the studies included did not evaluated the BDM combination as primary outcome. But they provided data that can be identified and analyzed as DBM combination. The following DBM combinations were identified: underweight and gestational diabetes mellitus, underweight and hypertension, overweight/obesity and anemia, overweight/obesity and vitamin D deficiency, overweight/obesity and vitamin B12 deficiency, and overweight/obesity and folate deficiency. Of the 62 studies, the most prevalent DBM combination was the coexistence of underweight and gestational diabetes mellitus evaluated in 34 (54%) articles [24, 25, 26, 30, 31, 32, 33, 36, 37, 38, 39, 42, 43, 44, 45, 46, 50, 51, 52, 53, 54, 55, 56, 57, 60, 61, 62, 63, 64, 65, 71, 75, 76, 79]; 18 (29%) overweight/obesity and anemia [26, 27, 34, 35, 36, 47, 48, 50, 55, 58, 61, 66, 73, 75, 77, 83, 84]; 15 (24%) underweight and hypertension [28, 32, 35, 37, 38, 40, 46, 54, 57, 61, 62, 71, 72, 78, 79]; 10 (16%) overweight/obesity and vitamin D deficiency [29, 36, 41, 67, 68, 69, 70, 80, 81, 82]; two (3.2%) overweight/obesity and vitamin B12 deficiency [36, 85]; and one (1.6%) overweight/obesity and folate deficiency [36, 85]. A summary of the characteristics and main results according to each DBM combination outcome is presented in Table 1 and Appendix S2.

**TABLE 1:** Characteristics of the studies included in the systematic review.

Characteristics	*N*	%
Study design		
Cohort	45	72.58%
Cross‐sectional	16	25.81%
Case control	1	1.61%
Location of data collection		
Health service	55	88.71%
Community	7	11.29%
Location (rural or urban)		
Urban	53	85.48%
Urban and rural	7	11.29%
Rural	2	3.23%
Simple size		
> 500	40	64.52%
≤ 500	22	35.48%
Methodological quality of the studies		
High	41	66.13%
Low	21	33.87%
Gestational trimester		
All	23	37.10%
First	14	22.58%
Third	7	11.29%
Second	5	8.06%
NR	5	8.06%
First and second	4	6.45%
Second and third	4	6.45%
Life cycle		
Adult	33	53.23%
Adult and adolescent	21	33.87%
NR	8	12.90%

The DBM combination of underweight and GDM or hypertension were identified primarily in studies conducted in Asia, including China, Bangladesh, Pakistan, India, South Korea, Malaysia, and Japan. The samples in these studies predominantly included pregnant adult women from urban areas. The prevalence of pregnant women with underweight and gestational diabetes mellitus ranged from 0.10% in Pakistan [55] and France [37] to 3.21% in Bangladesh [36]. The combination of underweight and hypertension was observed with a prevalence of 0.03% in Croatia [79] and 1% in India [35, 38, 46].

The DBM combination of overweight/obesity and micronutrient deficiency (anemia, vitamin D deficiency, vitamin B12 deficiency, and folate deficiency) showed wide variation in prevalence across studies. The pregnant women participating in the studies were mostly adults from urban areas, mostly Asian, with representation from South America and Africa. The DBM combination of overweight/obesity and anemia ranged from 0.27% in South Korea [26] to 40.40% in Nigeria [34]. The prevalence of overweight/obesity and vitamin D deficiency during pregnancy ranged from 5.06% in Taiwan [81] and 53.57% in Malaysia [68], respectively. Two studies evaluated the prevalence of overweight/obesity and vitamin B12 deficiency, reporting values of 2.01% in Bangladesh [36] and 12.79% in the United Kingdom [85]. The same studies also evaluated overweight and folate deficiency with values of 0.40% in Bangladesh [36] and 1.16% in the United Kingdom [85].

The DBM coexistence, considering several vitamin and folate deficiencies, was evaluated by only one study. Bhowmik et al. (2019) evaluated the impact of maternal BMI on nutritional status in early pregnancy, reporting outcomes, including anemia, gestational diabetes mellitus, hypertension, and deficiencies of vitamin D, B12, and folate [36]. The prevalence of coexisting overweight/obesity and anemia was 3.61%; underweight and gestational diabetes mellitus, 3.21%; underweight and hypertension, 0.16%; and vitamin D, B12, and folate deficiencies, 8.84%, 2.01%, 3.21%, and 0.40%, respectively [36].

### 3.4 Methodological Quality of Individual Studies

Of the 62 studies, 21 (33%) were considered as low risk of bias [25, 29, 37, 45, 51, 52, 58, 62, 63, 64, 65, 66, 67, 68, 69, 72, 73, 75, 79, 81, 84] and 41 (66%) as high risk [24, 26, 27, 28, 30, 31, 32, 33, 34, 35, 36, 38, 39, 40, 41, 42, 43, 44, 46, 47, 48, 49, 50, 53, 54, 55, 56, 57, 59, 60, 61, 70, 71, 74, 76, 77, 78, 80, 82, 83, 85], since they presented one or more items classified with the criteria “no” or “unclear” (Supporting Information Figures S6 and S7).

Overall, the cohort studies demonstrated a low risk of selection bias, with consistent participant recruitment, comparable exposure assessment between groups, and appropriate statistical analyses. However, some studies showed limitations in controlling for confounding factors and ensuring adequate participant follow‐up. The cross‐sectional studies showed adequate methodological quality, with clearly defined eligibility criteria, well‐described participants, and appropriate statistical analyses. Most studies did not identify or address potential confounding factors, which may have affected the validity of their findings. The complete risk of bias analysis is shown in Appendix S5. Supporting Information Figures S6 and S7 showed the risk of bias of selected cohort and cross‐sectional studies, respectively.

### 3.5 Meta‐Analysis of Results

**FIGURE 2:**
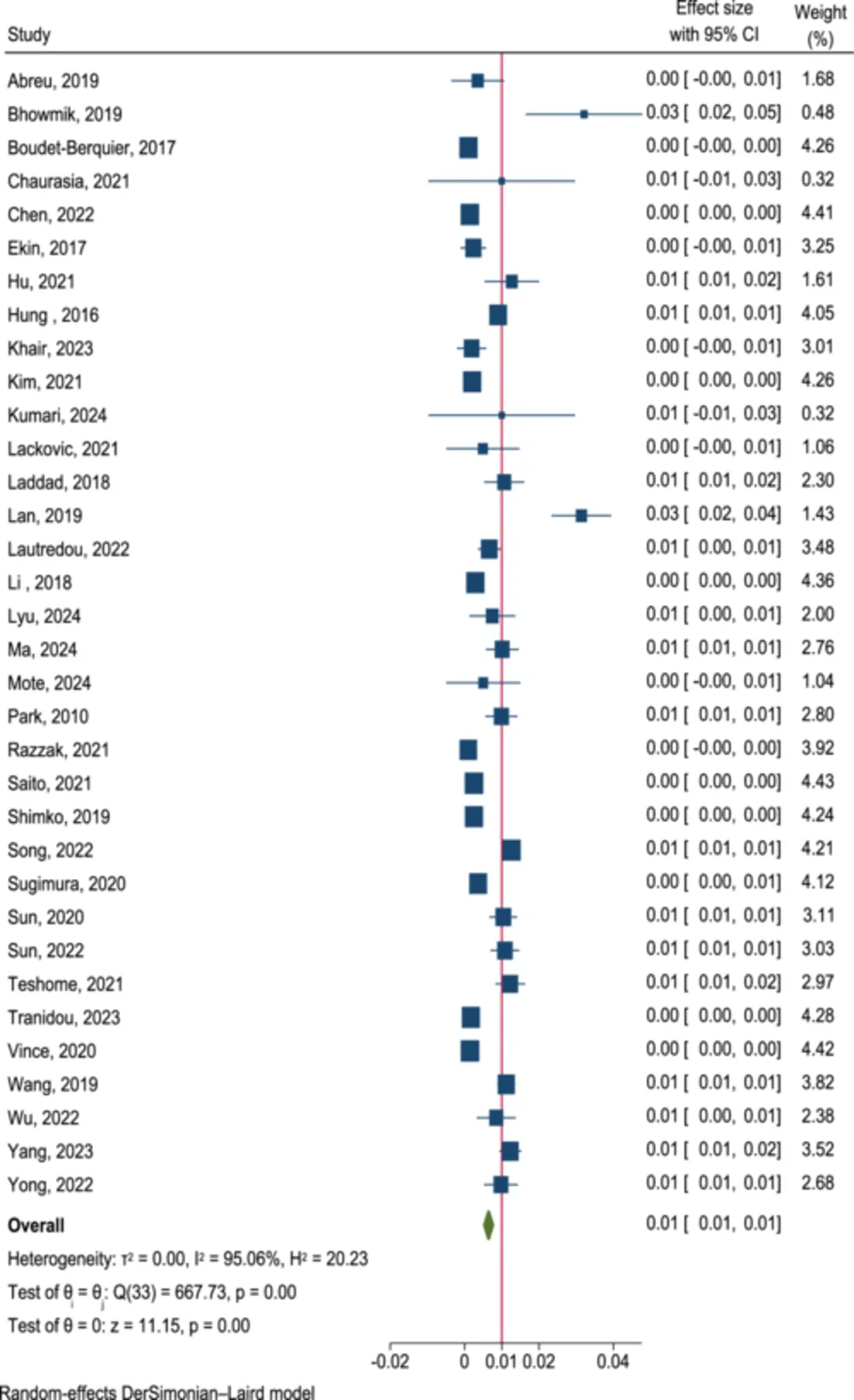
Prevalence of underweight and GDM in pregnant women.

Regarding the outcomes for the underweight‐related DBM, a low prevalence of this form of DBM was observed among the pregnant women evaluated in this review. The prevalence of DBM combination underweight and gestational diabetes mellitus was 1% (95% CI 01–01; *I* ^2^ = 95.06%, *n* = 34 studies (Figure 2)). And the prevalence of DBM combination underweight and hypertension was 0%, *n* = 15 studies (Supporting Information Figure S1).

The subgroup analysis was performed by continent, study design, gestational trimester, life cycle, and risk of bias, but no statistical differences were found in the overall prevalence in the DBM combination underweight and gestational diabetes mellitus. The subgroup analysis of DBM combination underweight and hypertension was not conducted due to the zero prevalence.

**FIGURE 3:**
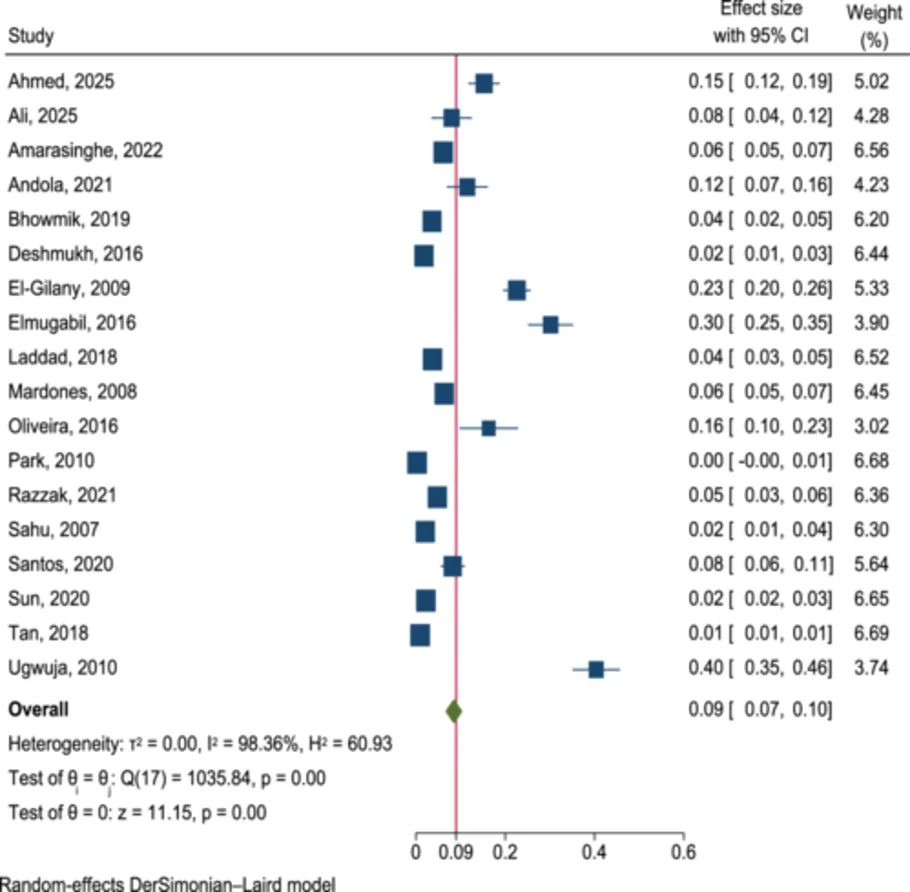
Prevalence of overweight/obesity and anemia in pregnant women.

**FIGURE 4:**
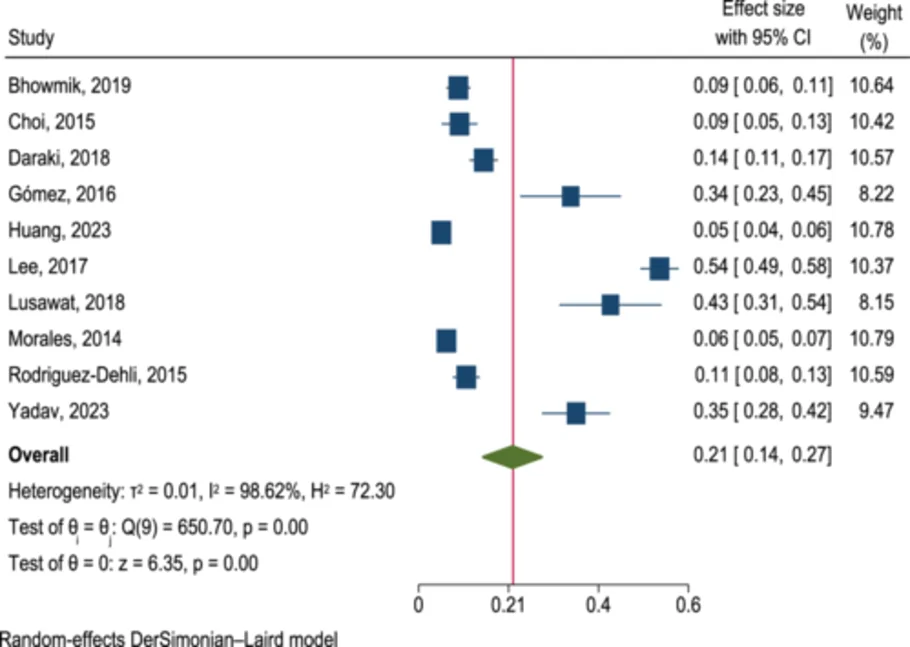
Prevalence of overweight/obesity and vitamin D deficiency in pregnant women.

When analyzing the overweight/obesity‐related DBM, a considerable prevalence was identified. The prevalence of pregnant women with DBM combination overweight/obesity and anemia was 9% (95% CI 07–10; *I* ^2^ – 98.36%, *n* = 18 studies (Figure 3)). And for the DBM combination with overweight/obesity and vitamin D deficiency the prevalence in pregnant women was 21% (95% CI 14–27; *I* ^2^ – 98.62%, *n* = 10 studies (Figure 4)).

The subgroup analysis related to DBM combination with anemia showed that Africa had a higher prevalence (29%; 95% CI: 13%–44%) than Asia (5%; 95% CI: 3%–5%) and South America (9%; 95% CI: 5%–12%). Regarding gestational trimester, studies that evaluated pregnant women in the third or second and third trimesters reported a higher prevalence than the all‐trimester group. The inclusion of pregnant adolescents in study samples results in greater DBM prevalence (13% 95% CI: 9%–18%).

The result for DBM combination with vitamin D deficiency showed a subgroup analyses with a higher prevalence in pregnant women from South America compared to Europe, and in pregnant women evaluated in the second and third trimesters compared to the all‐trimester group. The others subgroup analysis was performed, but no statistical differences were found in the overall prevalence related to vitamin D deficiency.

The overall prevalence of all DBM combination subgroups regarding all outcomes presents above is reported in Supporting Information Figures S2–S5.

**FIGURE 5:**
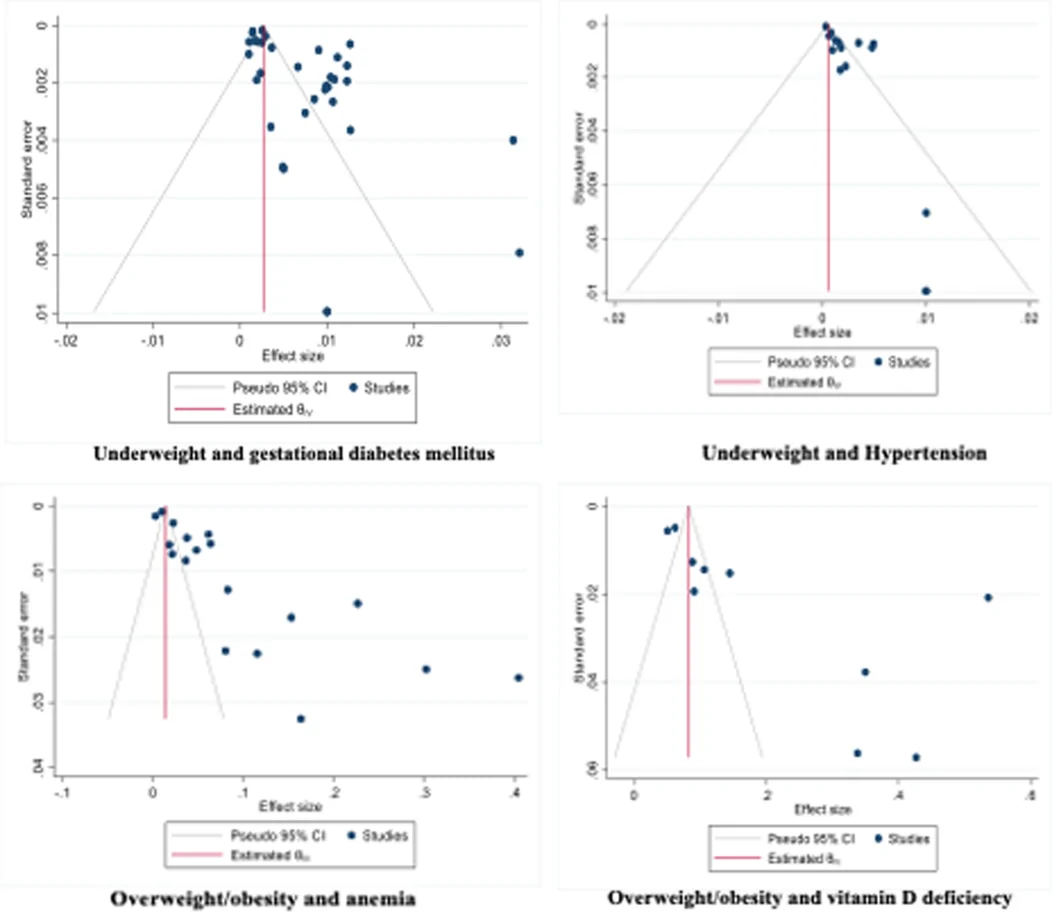
Funnel graph on the publication bias of studies according to each outcome.

Visual inspection of the funnel plot (Figure 5) suggested asymmetry, and Egger's regression test was statistically significant (*p* < 0.001). Nevertheless, these findings should not be interpreted as conclusive evidence of publication bias, as funnel plot asymmetry in meta‐analyses of prevalence may result from several factors, including substantial between‐study heterogeneity, methodological differences, small‐study effects, and the bounded nature of proportion estimates.

At last, using the GRADE approach for prevalence studies, the certainty of the evidence was judged to be low quality (Table 2), indicating that there is limited confidence in the pooled prevalence estimates because of important limitations across one or more GRADE domains, including inconsistency and publication bias, as applicable. Inconsistency was considered very serious when *I* ^2^ presents values more than 40% and publication bias was rated as serious because visual inspection of the funnel plots suggested marked asymmetry and Egger's regression test was statistically significant.

**TABLE 2:** GRADE evidence profile for the prevalence of DBM according to each outcome.

Outcomes	Number of studies	Risk of bias^a^	Inconsistency^b^	Indirectness^c^	Imprecision^d^	Publication bias^e^	Certainty
Prevalence of underweight and gestational diabetes mellitus	34	Not serious	Very serious	Not serious	Not serious	Serious	⊕⊕◯◯ Low
Prevalence of overweight/obesity and anemia	18	Not serious	Very serious	Not serious	Not serious	Serious	⊕⊕◯◯ Low
Prevalence of underweight and hypertension	16	Not serious	Very serious	Not serious	Not serious	Serious	⊕⊕◯◯ Low
Prevalence of overweight/obesity and vitamin D deficiency	10	Not serious	Very serious	Not serious	Not serious	Serious	⊕⊕◯◯ Low

^a^Risk of bias assessed using JBI. No downgrade, considering appropriate sampling method and statistical analyses in more than 75% of studies. ^b^Heterogeneity was considered important when *I* ^2^ presents values more than 40%. Downgrade 1 level for inconsistency because *I* ^2^ = 95.06% (studies with prevalence of underweight and GDM), *I* ^2^ = 98.36% (studies with prevalence of overweight/obesity and anemia in pregnant women), *I* ^2^ = 83.71% (studies with prevalence of underweight and hypertension) and *I* ^2^ = 98.62% (studies with prevalence of overweight/obesity and vitamin D deficiency in pregnant women). ^c^No downgrade for indirectness because less than 25% of studies did not use valid and reliable methods for data collection. ^d^No downgrade for imprecision because more than 75% of studies a sample size > 500. ^e^Publication bias was considered when the significance of *p* < 0.05. Downgrade 1 level, because *p* = 0.0000 (studies with prevalence of underweight and GDM), *p* = 0.0000 (studies with prevalence of overweight/obesity and anemia in pregnant women), *p* = 0.0015 (studies with prevalence of underweight and hypertension), and *p* = 0.0000 (studies with prevalence of overweight/obesity and vitamin D deficiency in pregnant women).

## 4 Discussion

Although some studies have evaluated individual DBM combination in pregnancy groups, we believe this is the first systematic review conducted with a meta‐analysis on the prevalence of individual DBM in pregnant women following the concept defined by the WHO (2017) [1]. Four DMB coexistences were highlighted, providing sufficient data for quantitative synthesis: a high prevalence of overweight/obesity with micronutrient deficiency (overweight/obesity and anemia; overweight/obesity and vitamin D deficiency), and a low prevalence of underweight and gestational diabetes mellitus, as well as underweight and hypertension. The worldwide prevalence of DBM during pregnancy varies significantly by region [86]. It represents an emergent public health issue since nearly one‐third of the adult population suffers from at least one form of malnutrition [87], and obesity has doubled over the past 30 years, while obesity in LMICs has tripled over the past 20 years [88]. Factors such as wealth, education, and dietary intake are key determinants, with wealthy and highly educated women often at higher risk of overweight and obesity [89]. At the same time, food insecurity increases the chances of both excessive weight gain and inadequate weight gain during pregnancy, and contributes to a broader DBM during pregnancy [90].

The high prevalence of DBM combination in overweight/obesity nutritional status found is aligned with the prevalence of overweight and obesity, which keeps increasing globally [91], since most countries have undergone fast changes in dietary patterns and lifestyle [92]. About 85% os the pregnant women included in this review reside in an urban area and one‐third of the studies included only adult women. Populations, particularly in urban areas, are increasingly exposed to foods that are high in sugar, energy‐dense, and low in micronutrients. Additionally, unhealthy food systems, social vulnerability, food marketing, income, and the global syndemic framework of obesity, undernutrition, and climate change interact and affect pregnant women's health [93].

Overweight and obesity complicate up to two‐thirds of pregnancies, and increase risk of gestational diabetes, hypertension, pre‐eclampsia, and maternal and fetal death [94]. The WHO estimates that anemia is a major global public health concern affecting roughly 37% of pregnant women worldwide, driven primarily by iron deficiency, increased nutrient demands, and socioeconomic disparities [95]. Although these conditions may seem unrelated and their presence almost antagonistic, they do coexist, and Launbo et al. (2022) estimate their prevalence to range from 0% to 40.3% during pregnancy [96]. Our study reported a 9% global prevalence of this DBM combination, showing how women experience multiple nutritional challenges concurrently. Given the higher prevalence of concomitant overweight/obesity and anemia found among pregnant women on the African continent, this subgroup analysis suggests that socioeconomic context, urban residence, diet quality, and access to health services may influence the prevalence of these conditions [97, 98, 99]. This result represents a paradoxical situation that corroborates the nutritional overlap observed worldwide. The urban and adult profile of the pregnant women included may explain the higher prevalence found since the increased consumption of ultra‐processed foods in LMICs, which, in addition to contributing to excessive weight gain, are also linked to reduced intake of micronutrients such as iron and folic acid, which are important during pregnancy and for reducing anemia [100]. Also, the variability observed in the diagnosis of anemia, with most studies adopting the WHO criterion, whereas others used alternative definitions, such as hemoglobin < 10.5 g/dL or iron‐deficiency anemia based on combined hemoglobin and serum ferritin measurements contributes to the high heterogeneity presented.

The 21% prevalence of DBM combined overweight/obesity and vitamin D deficiency in pregnant women observed in this meta‐analysis highlights the impact of excessive adipose tissue during pregnancy. The studies included in this systematic review did not aim to estimate the prevalence of this BDM combined but focused on outcomes associated with vitamin D deficiency, such as preeclampsia, gestational diabetes mellitus, and low birth weight [29]. The substantial heterogeneity observed across studies is likely explained by important clinical and methodological differences among the included studies. The studies were conducted in diverse geographical regions, including Asia, Europe, and South America, where differences in sunlight exposure, dietary habits, food fortification policies, ethnicity, and socioeconomic conditions may influence both obesity and vitamin D status. Furthermore, the included studies adopted different criteria to classify nutritional status, using either pre‐pregnancy or gestational BMI and different BMI cut‐off values, reflecting both WHO and population‐specific recommendations. Likewise, considerable variation was observed in the definition of vitamin D deficiency, with cut‐off values ranging from < 10 to < 20 ng/mL and < 30 to < 37.7 nmol/L, as well as differences in the biomarkers and laboratory methods used to assess serum vitamin D concentrations.

Additional sources of heterogeneity include differences in gestational trimester at assessment (first, second, third, all trimesters, or mixed populations), study design (cohort and cross‐sectional), sample size (ranging from 71 to 2489 participants), and methodological quality also contributes to justify the the substantial between‐study heterogeneity observed and should be considered when interpreting the pooled prevalence estimates for both DBM combinations.

Furthermore, the low prevalence of gestational diabetes mellitus with underweight was somewhat expected, as the literature robustly establishes the association of GDM with overweight and obesity [56, 101]. Although underweight during pregnancy is a protective factor against GDM, the occurrence of GDM can increase significantly as the pregnant woman's age advances, reinforcing the importance of actions to limit excessive weight gain in pregnant women to prevent the occurrence of GDM [102]. Evaluations of the coexistence of overweight/obesity with vitamin B12 or folate deficiency were limited, making it impossible to conduct the meta‐analysis. However, it is noteworthy that the combination of nutritional deficiency and excess weight warrants further study, given its impact on maternal‐child health. The higher prevalence of ultra‐processed food consumption, ranging between 18.2% and 25.4% of daily kcal, in pregnant women may justify this scenario [103, 104, 105]. Biete et al. (2023) showed, using a multivariate model, that healthy pregnant women in the highest quintile of ultra‐processed food intake had lower iron and folate intake, reinforcing the need for adequate macro‐ and micronutrient intake to strengthen the nutritional quality of pregnant women's diets [100].

Finally, although funnel plots and Egger's regression test were performed to explore potential small‐study effects, these methods have recognized limitations in meta‐analyses of prevalence. Therefore, any observed asymmetry should not be interpreted as conclusive evidence of publication bias, as it may also reflect heterogeneity or other characteristics of prevalence data. According to the GRADE system, the grouping of studies on the prevalence of DBM combination in pregnant women provided low‐quality evidence. However, it is important to note that estimating the prevalence of outcomes in the review requires evaluating observational studies, and the GRADE system considers them to have a “low‐quality” level of evidence. Therefore, a GRADE result does not necessarily indicate a limitation in the evidence, but often indicates a limitation in the body of evidence itself, such as having very few studies, small sample sizes, or few events.

The identification of various coexistences of DBM highlights the need to consider more than just isolated approaches to treating overweight/obesity, underweight, anemia, nutrient deficiencies, and chronic noncommunicable diseases. Also, considering that the United Nations General Assembly proclaimed the Decade of Action for Nutrition (2016–2025), extended to 2030 and the goal “to end malnutrition in all its forms” [106], the findings of this review contribute to extol the relevance of more effective public policies focused on the nutrition status of pregnant women, aiming to minimize the harm of malnutrition to maternal‐child health.

The study's strengths include its comprehensive and up‐to‐date approach to the issue of the individual DBM combination during pregnancy, filling a gap in data presentation that currently focuses on population or household‐level data with mixed groups. It is worth noting that the subgroup analysis also allowed for a more detailed understanding of the factors that influence the DBM. Furthermore, the conceptual alignment with the WHO definition lends robustness to the study and provides important data to inform decision‐making regarding the growing impact of DBM on maternal and child health. Clinical and methodological differences across studies, including variations in BMI classification during pregnancy and in the biomarkers and diagnostic cut‐off values used for micronutrient deficiencies, may have contributed to the observed heterogeneity and limited the comparability of the pooled prevalence estimates. Furthermore, it is noteworthy that most of the studies analyzed did not report DBM combination as an outcome but were included in the review when they provided the necessary data for this analysis. So, we reinforce the need for studies focusing on DBM with robust designs to more deeply explore the mechanisms involved and the risk factors underlying each condition of concomitant malnutrition in pregnant women, especially at the individual level. The scarcity of studies with standardized methodologies and representative samples underscores the importance of conducting more research that explores, in addition to prevalence, risk factors, and outcomes, the individual DBM combination in pregnant women. Finally, the use of the DerSimonian–Laird estimator, which may underestimate between‐study variance under substantial heterogeneity, although it remains a widely used approach for random‐effects meta‐analysis.

These findings offer valuable insights for improving clinical practices and public policies by highlighting the prevalence of the main forms of DBM combination at the individual level among pregnant women and by discussing ways to prevent and combat the various forms of malnutrition during this critical period for maternal and child health.

## 5 Conclusion

This systematic review and meta‐analysis showed that the DBM combination is globally present among pregnant women. The prevalence of overweight/obesity and vitamin D deficiency was the most prevalent DBM (21%), followed by the prevalence of overweight/obesity and anemia (9%), and a prevalence of 1% among pregnant women with underweight and gestational diabetes mellitus. The findings demonstrate that the DBM represents a significant challenge for pregnant women, considering the harm to mother‐child health and association with adverse outcomes.

## Author Contributions

Amanda Biete and Camila Biete made substantial contributions in the design of the study concept, performed the searches, selection of studies, data extraction, analysis, and interpretation, in addition to writing the article and review and approval the final version to be submitted. Vivian S. S. Gonçalves and Erika S. O. Patriota contributed to the design of the study concept, as well as contributed to the statistical analysis, writing the article, and approving the final version to be submitted. Nathalia Pizato contributed to the design of study concept, data analysis and interpretation, writing the article, review, and approval the version to be submitted.

## Conflicts of Interest

The authors declare no conflicts of interest.

## Supporting information


**Figure S1**: Prevalence of underweight and hypertension in pregnant women.

**Figures S2 to S5:** Prevalence of double burden of nutrition in pregnant women for subgroups.

**Figures S6 and S7:** Risk of Bias of Selected Cohort Studies and Cross‐Sectional Studies (The Joanna Briggs Institute Critical Appraisal checklist).

**Appendix S1:** PRISMA checklist.

**Appendix S2:** Summary of characteristics of studies.

**Appendix S3:** Search strategy.

**Appendix S4:** Excluded articles and reasons for exclusion.

**Appendix S5:** Risk of bias for each study assessed by Joanna Briggs Institute critical appraisal checklist.

## Data Availability

The data that support the findings of this study are available in the Supporting Material of this article.
