# Supplementary material for: Double Burden of Malnutrition Prevalence in Pregnant Women: Systematic Review and Meta‐Analysis
Source: J Hum Nutr Diet. 2026 Sep 17;39(5):e70347. doi: 10.1111/jhn.70347 (PMC13586418; doi:10.1111/jhn.70347)
Supplement: Supplementary file 1 [file JHN-39-0-s001.docx]

Appendix S3. Database search strategy.

| **DATABASE** | **SEARCH** (July, 19, 2025) |
| --- | --- |
| **Pubmed (Medline)** | (((Pregnancy OR "Pregnant women" OR Gravidity OR Pregnant OR Gravid OR Gestation) AND (anemia OR anaemia OR "iron deficiency anemia" OR "iron deficiency anaemia" OR "Iron deficiency" OR "Iron-Deficiency Anemia" OR "Maternal anemia" OR "Deficiency Diseases" OR Avitaminosis OR "Nutritional Deficiencies" OR "Vitamin Deficiencies" OR Hypertension OR Diabetes OR Hyperglycemia)) AND ("Double burden of malnutrition" OR "Nutritional Deficiency" OR "Nutritional Deficiencies" OR "maternal obesity" OR "Obesity in Pregnancy" OR "gestational weight gain" OR "maternal overweight" OR Undernutrition OR Underweight OR Undernourished OR Stunting OR Wasting)) AND ("Observational Study" OR "Cohort study" OR "Longitudinal study" OR "Follow-up study" OR Cohort OR Longitudinal OR Prospective OR Retrospective OR "Incidence study" OR Follow-up OR "Prevalence study" OR Prevalence OR "Cross-Sectional Study" OR Cross-Sectional OR frequency) |
| **Scopus** | (((Pregnancy OR "Pregnant women" OR Gravidity OR Pregnant OR Gravid OR Gestation) AND (anemia OR anaemia OR "iron deficiency anemia" OR "iron deficiency anaemia" OR "Iron deficiency" OR "Iron-Deficiency Anemia" OR "Maternal anemia" OR "Deficiency Diseases" OR Avitaminosis OR "Nutritional Deficiencies" OR "Vitamin Deficiencies" OR Hypertension OR Diabetes OR Hyperglycemia)) AND ("Double burden of malnutrition" OR "Nutritional Deficiency" OR "Nutritional Deficiencies" OR "maternal obesity" OR "Obesity in Pregnancy" OR "gestational weight gain" OR "maternal overweight" OR Undernutrition OR Underweight OR Undernourished OR Stunting OR Wasting)) AND ("Observational Study" OR "Cohort study" OR "Longitudinal study" OR "Follow-up study" OR Cohort OR Longitudinal OR Prospective OR Retrospective OR "Incidence study" OR Follow-up OR "Prevalence study" OR Prevalence OR "Cross-Sectional Study" OR Cross-Sectional OR frequency) |
| **Embase** | (((Pregnancy OR "Pregnant women") AND (anemia OR anaemia OR "iron deficiency anemia" OR "Iron deficiency" OR "Iron-Deficiency Anemia" OR "Maternal anemia" OR "Deficiency Diseases" OR Avitaminosis OR "Nutritional Deficiencies" OR "Vitamin Deficiencies" OR Hypertension OR Diabetes OR Hyperglycemia)) AND ("Double burden of malnutrition" OR "Nutritional Deficiency" OR "Nutritional Deficiencies" OR "maternal obesity" OR "Obesity in Pregnancy" OR "gestational weight gain" OR "maternal overweight" OR Undernutrition OR Underweight OR Undernourished OR Stunting OR Wasting)) AND ("Observational Study" OR "Cohort study" OR "Longitudinal study" OR "Follow-up study" OR Cohort OR Longitudinal OR Prospective OR Retrospective OR "Incidence study" OR Follow-up OR "Prevalence study" OR Prevalence OR "Cross-Sectional Study" OR Cross-Sectional OR frequency) |
| **Web of Science** | (((Pregnancy OR "Pregnant women" OR Gravidity OR Pregnant OR Gravid OR Gestation) AND (anemia OR anaemia OR "iron deficiency anemia" OR "iron deficiency anaemia" OR "Iron deficiency" OR "Iron-Deficiency Anemia" OR "Maternal anemia" OR "Deficiency Diseases" OR Avitaminosis OR "Nutritional Deficiencies" OR "Vitamin Deficiencies" OR Hypertension OR Diabetes OR Hyperglycemia)) AND ("Double burden of malnutrition" OR "Nutritional Deficiency" OR "Nutritional Deficiencies" OR "maternal obesity" OR "Obesity in Pregnancy" OR "gestational weight gain" OR "maternal overweight" OR Undernutrition OR Malnourishments OR Underweight OR Undernourished OR Stunting OR Wasting)) AND ("Observational Study" OR "Cohort study" OR "Longitudinal study" OR "Follow-up study" OR Cohort OR Longitudinal OR Prospective OR Retrospective OR "Incidence study" OR Follow-up OR "Prevalence study" OR Prevalence OR "Cross-Sectional Study" OR Cross-Sectional OR frequency) |
| **Lilacs** | (((Pregnancy OR "Pregnant women" OR Gravidity OR Pregnant OR Gravid OR Gestation) AND (anemia OR anaemia OR "iron deficiency anemia" OR "iron deficiency anaemia" OR "Iron deficiency" OR "Iron-Deficiency Anemia" OR "Maternal anemia" OR "Deficiency Diseases" OR Avitaminosis OR "Nutritional Deficiencies" OR "Vitamin Deficiencies" OR Hypertension OR Diabetes OR Hyperglycemia)) AND ("Double burden of malnutrition" OR "Nutritional Deficiency" OR "Nutritional Deficiencies" OR "maternal obesity" OR "Obesity in Pregnancy" OR "gestational weight gain" OR "maternal overweight" OR Undernutrition OR Malnourishments OR Underweight OR Undernourished OR Stunting OR Wasting)) AND ("Observational Study" OR "Cohort study" OR "Longitudinal study" OR "Follow-up study" OR Cohort OR Longitudinal OR Prospective OR Retrospective OR "Incidence study" OR Follow-up OR "Prevalence study" OR Prevalence OR "Cross-Sectional Study" OR Cross-Sectional OR frequency) |
| **Scielo** | (((Pregnancy OR "Pregnant women" OR Gravidity OR Pregnant OR Gravid OR Gestation) AND (anemia OR anaemia OR "iron deficiency anemia" OR "iron deficiency anaemia" OR "Iron deficiency" OR "Iron-Deficiency Anemia" OR "Maternal anemia" OR "Deficiency Diseases" OR Avitaminosis OR "Nutritional Deficiencies" OR "Vitamin Deficiencies" OR Hypertension OR Diabetes OR Hyperglycemia)) AND ("Double burden of malnutrition" OR "Nutritional Deficiency" OR "Nutritional Deficiencies" OR "maternal obesity" OR "Obesity in Pregnancy" OR "gestational weight gain" OR "maternal overweight" OR Undernutrition OR Malnourishments OR Underweight OR Undernourished OR Stunting OR Wasting)) AND ("Observational Study" OR "Cohort study" OR "Longitudinal study" OR "Follow-up study" OR Cohort OR Longitudinal OR Prospective OR Retrospective OR "Incidence study" OR Follow-up OR "Prevalence study" OR Prevalence OR "Cross-Sectional Study" OR Cross-Sectional OR frequency) |
| **Scielo** | ((Gravidez OR "Mulheres grávidas" OR Gestação) AND (anemia OR "anemia por deficiência de ferro" OR "Deficiência de ferro" OR "Maternal anemia" OR Avitaminose OR "Deficiências nutricionais" OU "Deficiências vitamínicas" OR Hipertensão OR Diabetes OR Hiperglicemia)) AND ("Dupla carga de má nutrição" OR "Deficiência nutricional" OR "Deficiências nutricionais" OR "obesidade materna" OR "ganho de peso gestacional" OR "sobrepeso materno" OR Desnutrição OR Retardo de crescimento) |
| **Scielo** | ((Embarazo OR "Mujeres embarazadas" OR Embarazo) AND (anemia OR "anemia por deficiencia de hierro" OR "Deficiencia de hierro" OR "Anemia materna" OR Avitaminosis OR "Deficiencias nutricionales" OR "Deficiencias de vitaminas" OR Hipertensión OR Diabetes OR Hiperglucemia)) AND ("Doble carga de desnutrición" OR "Deficiencia nutricional" OR "Deficiencias nutricionales" OR "obesidad materna" OR "aumento de peso gestacional" OR "sobrepeso materno" OR Desnutrición OR Bajo peso OR Insuficiencia del crecimiento) |
| **Proquest** | ((Pregnancy OR Pregnant) AND ("Double burden of malnutrition" OR "Nutritional deficiency") AND ("Maternal anemia" OR Hypertension OR Diabetes OR Obesity)) |
| **Google Scholar** | ((Pregnancy OR Pregnant) AND ("Double burden of malnutrition" OR "Nutritional deficiency" OR Undernutrition OR Underweight OR "Vitamin deficiency") AND ("Maternal anemia" OR Hypertension OR Diabetes OR Obesity)) |
| **Google Acadêmico** | ((Gestação OR Gestante) AND ("Dupla carga de má nutrição" OR "Deficiência nutricional" OR Desnuitrição OR "Baixo peso" OR "Deficiência de vitamina") AND ("Anemia materna" OR Hipertensão OR Diabetes OR Obesidade)) |
