# Supplementary material for: Double Burden of Malnutrition Prevalence in Pregnant Women: Systematic Review and Meta‐Analysis
Source: J Hum Nutr Diet. 2026 Sep 17;39(5):e70347. doi: 10.1111/jhn.70347 (PMC13586418; doi:10.1111/jhn.70347)
Supplement: Supplementary file 3 [file JHN-39-0-s003.docx]

**Appendix S2 –** Sumary of characteristics of studies

**Table S1 -** Summary of characteristics of included studies that presented data on the prevalence of underweight and gestational diabetes mellitus coexistence

| **Author/Year** | **Country** | **Continent** | **Study Design** | **Gestational Trimester** | **Life Cicle** | **BMI** | **GDM Diagnostic Criteria** | **Sample**  **Size** | **Risk Of Bias (High Or Low)** | **Prevalence Of Underweight And Gestational Diabetes Mellitus (%)** |
| --- | --- | --- | --- | --- | --- | --- | --- | --- | --- | --- |
| Abreu, 2019 | Brazil | South America | Cross-sectional | Third | Adult | Pre-gestational BMI Underweight <18.5 kg/m2 (IOM, 2009) | TOTG 75 g glucose 24 – 28 weeks (IADPSG) International Association of Diabetes and Pregnancy Study Group (IADPSG) | 283 | Low | 0,35% (0.017 – 1.73) |
| Bhowmik, 2019 | Bangladesh | Asia | Cohort | First | Adult | Gestational BMI Underweight <18.5 kg/m2 (WHO, 2000) | TOTG 75 g glucose 24 – 28 weeks (IADPSG) | 498 | High | 3,21% (1.914 – 5.056) |
| Boudet-Berquier, 2017 | France | Europe | Cohort | NR | Adult | Pre-gestational BMI Underweight <18.5 kg/m2 (WHO, 2000) | TOTG 75 g glucose 24 – 28 weeks (IADPSG) | 3368 | Low | 0,10% (0.023 – 0.254) |
| Chaurasia, 2021 | India | Asia | Cohort | First | Adult and adolescent | Pre-gestational BMI Underweight <18.5 kg/m2 (IOM, 2009) | NR | 100 | High | 1,00% (0.05 – 4.832) |
| Chen, 2022 | Finland | Europe | Cohort | NR | NR | Pre-gestational BMI Underweight <18.5 kg/m2 (NR Criteria) | TOTG 75 g glucose 24 – 28 weeks (IADPSG) | 1097753 | High | 0,14% (0.100 – 0.194) |
| Ekin, 2017 | Turkey | Asia | Cohort | NR | Adult and adolescent | Pre-gestational BMI Underweight <18.5 kg/m2 (IOM, 2009) | TOTG 75 g glucose 24 – 28 weeks (IADPSG) | 849 | High | 0,24% (0.039 – 0.776) |
| Hu, 2021 | China | Asia | Cohort | Second | NR | Pre-gestational BMI Underweight <18.5 kg/m2 (HAPO, 2009) | TOTG 75 g glucose 24 – 28 weeks (IADPSG) | 945 | High | 1,27% (0.689 – 2.149) |
| Hung, 2016 | Taiwan | Asia | Cohort | All | Adult and adolescent | Pre-gestational BMI Underweight <18.5 kg/m2 (IOM, 2009) | NR | 12064 | High | 0,90% (0.746 – 1.085) |
| Khair, 2023 | United Arab Emirates | Asia | Cross-sectional | All | Adult | Pre-gestational BMI Underweight <18.5 kg/m2 (IOM, 2009) | NR | 526 | High | 0,19% (0.002 – 0.933) |
| Kim, 2021 | South Korea | Asia | Cohort | All | NR | Gestational BMI Underweight <18.5 kg/m2 (WHO, 2000) | NR | 6331 | Low | 0,21% (0.114 – 0.342) |
| Kumari, 2024 | India | Asia | Cohort | All | Adult | Pre-gestational BMI Underweight <18.5 kg/m2 (IOM, 2009) | TOTG 75 g glucose 24 – 28 weeks (IADPSG) | 100 | High | 1% (0.050 – 4.83) |
| Lackovic, 2021 | Serbia | Europe | Cohort | First and Second | Adult | Pre-gestational BMI Underweight <18.5 kg/m2 (IOM, 2009) | TOTG 75 g glucose 24 – 28 weeks (IADPSG) | 203 | High | 0,49% (0.024 – 2.405) |
| Laddad, 2018 | India | Asia | Cohort | First | Adult | Pre-gestational BMI Underweight <19.9 kg/m2 (NR Criteria) | NR | 1500 | High | 1.07% (0.632 – 1.69) |
| Lan, 2019 | China | Asia | Cohort | First and Second | Adult | Pre-gestational BMI Underweight <18.5 kg/m2 (Chinese Criteria) | TOTG 75 g glucose 24 – 28 weeks (IADPSG) | 1910 | High | 3,14% (2.427 – 3.997) |
| Lautredou, 2022 | France | Europe | Cohort | Second and third | Adult | Pre-gestational BMI Underweight <18.5 kg/m2 (IOM, 2009) | TOTG 75 g glucose 24 – 28 weeks (IADPSG) | 3162 | High | 0,66% (0.422 – 0.996) |
| Li, 2018 | China | Asia | Cohort | All | Adult | Pre-gestational BMI Underweight <18.5 kg/m2 (Chinese Criteria) | TOTG 75 g glucose 24 – 28 weeks (IADPSG) | 19962 | High | 0,29% (0.222 – 0.373) |
| Lyu, 2024 | China | Asia | Cohort | All | Adult | Pre-gestational BMI Underweight <18.5 kg/m2 (WHO, 2000) | TOTG 75 g glucose 24 – 28 weeks (IADPSG) | 802 | Low | 0,75% (0.303 – 1.55) |
| Ma, 2024 | China | Asia | Cohort | All | NR | Pre-gestational BMI Underweight <18.5 kg/m2 (IOM, 2009) | TOTG 75 g glucose 24 – 28 weeks (IADPSG) | 2174 | Low | 1,01% (0.638 – 1.471) |
| Mote, 2024 | India | Asia | Cohort | First | NR | Pre-gestational BMI Underweight <18.5 kg/m2 (IOM, 2009) | TOTG 75 g glucose 24 – 28 weeks (IADPSG) | 200 | High | 0,50% (0.024 – 2.441) |
| Park, 2010 | South Korea | Asia | Cohort | NR | NR | Pre-gestational BMI Underweight <18.5 kg/m2 (WHO, 2000) | TOTG 75 g glucose 24 – 28 weeks (IADPSG) | 2311 | High | 0,99% (0.638 – 1.471) |
| Razzak, 2021 | Pakistan | Asia | Cohort | All | Adult and adolescent | Pre-gestational BMI Underweight <18.5 kg/m2 (WHO, 2000) | NR | 1000 | High | 0,10% (0.005 – 0.492) |
| Saito, 2021 | Japan | Asia | Cohort | All | Adult | Pre-gestational BMI Underweight <18.5 kg/m2 (Japanese Criteria) | TOTG 75 g glucose 24 – 28 weeks (IADPSG) | 85228 | High | 0,25% (0.221 – 0.288) |
| Shimko, 2019 | Slovakia | Europe | Cohort | All | Adult | Pre-gestational BMI Underweight <18.5 kg/m2 (IOM, 2009) | TOTG 50 g glucose 24 – 28 weeks (NR Criteria) | 7122 | High | 0,25% (0.1545 – 0.391) |
| Song, 2022 | China | Asia | Cohort | All | Adult | Pre-gestational BMI Underweight <18.5 kg/m2 (Chinese Criteria) | 5.1 mmol/L for fasting serum glucose, 10.0 mmol/L for 1-h serum glucose, or 8.5 mmol/L for 2-h serum glucose | 29415 | High | 1,26% (1.141 – 1.398) |
| Sugimura, 2020 | Japan | Asia | Cohort | All | Adult | Pre-gestational BMI Underweight <18.5 kg/m2 (IOM, 2009) | Fasting blood glucose level of ≥92 mg/dL, blood glucose level at 1 h of ≥180 mg/dL, or blood | 6066 | High | 0,36% (0.233 – 0.539) |
| Sun, 2022 | China | Asia | Cohort | All | Adult and adolescent | Pre-gestational BMI Underweight <18.5 kg/m2 (WHO, 2004) | NR | 3043 | High | 1,08% (0.769 – 1.502) |
| Sun, 2020 | China | Asia | Cohort | All | Adult and adolescent | Pre-gestational BMI Underweight <18.5 kg/m2 (WHO,2004) | ≥5.1 mmol/L, 1 h plasma glucose levels ≥10.0 mmol/L, 2 h glucose levels ≥8.5 mmol/L after overnight fasting with a 75 g glucose load according to the WHO 2013 diag-nostic criteria | 3172 | High | 1,04% (0.729 – 1.441) |
| Teshome, 2021 | China | Asia | Cohort | First | Adult | Pre-gestational BMI Underweight <18.5 kg/m2 (Chinese Criteria) | TOTG 75 g glucose 24 – 28 weeks (IADPSG) | 3253 | High | 1,23% (0.891 – 1.654) |
| Tranidou, 2023 | Greece | Europe | Cohort | First and Second | Adult | Pre-gestational BMI Underweight <18.5 kg/m2 (IOM, 2009) | Fasting blood glucose ≥92 mg/dL, blood glucose concen-tration at one hour post OGTT ≥ 180 mg/dL, and/or blood glucose concentration 2 h post OGTT ≥ 153 mg/dL. | 5948 | High | 0,17% (0.729 – 1.441) |
| Vince, 2020 | Croatia | Europe | Cross-sectional | All | Adult and adolescent | Pre-gestational BMI Underweight <18.5 kg/m2 (IOM, 2009) | TOTG 75 g glucose 24 – 28 weeks (IADPSG) | 32051 | Low | 0,15% (0.109 – 0.193) |
| Wang, 2019 | China | Asia | Cohort | Second and third | Adult | Pre-gestational BMI Underweight <18.5 kg/m2 (IOM, 2009) | TOTG 75 g glucose 24 – 28 weeks (China Criteria) | 8926 | Low | 1,12% |
| Wu, 2022 | China | Asia | Cohort | Second | NR | Pre-gestational BMI Underweight <18.5 kg/m2 (WHO) | Fasting blood glucose ≥ 5.1 mmol/L (millimoles per liter). Blood glucose 1 hour after the 75g oral glucose tolerance test (OGTT) ≥ 10.0 mmol/L. Blood glucose 2 hours after the 75g OGTT ≥ 8.5 mmol/L | 1289 | Low | 0,85% |
| Yang, 2023 | China | Asia | Cohort | All | Adult | Pre-gestational BMI Underweight <18.5 kg/m2 (Chinese Criteria) | Fasting blood glucose ≥ 5.1 mmol/L (millimoles per liter)  Blood glucose 1 hour after the 75g oral glucose tolerance test (OGTT) ≥ 10.0 mmol/L. Blood glucose 2 hours after the OGTT with 75g ≥ 8.5 mmol/L | 6174 | Low | 1,23% |
| Yong, 2022 | Malaysia | Asia | Cohort | Second | Adult | Pre-gestational BMI Underweight <18.5 kg/m2 (WHO,1995) | Fasting blood glucose (FPG) ≥ 5.6 mmol/L.  Plasma glucose 2 hours after testing (2hPG) ≥ 7.8 mmol/L. | 1945 | Low | 0,98% |

Legend: NR – Not Reported

**Table S2 –** Summary of characteristics of included studies that presented data on the prevalence of underweight and gestational hypertension coexistance

| **Author/Year** | **Country** | **Continent** | **Study Design** | **Gestational Trimester** | **Life Cicle** | **BMI** | **Hypertension Diagnostic Criteria** | **Sample Size** | **Risk of Bias (High or Low)** | **Prevalence of underweight and gestational hypertension (%)** |
| --- | --- | --- | --- | --- | --- | --- | --- | --- | --- | --- |
| Andola, 2021 | India | Asia | Cohort | Third | Adult and adolescent | Pre-gestational BMI Underweight <18.5 kg/m2 (OMS) | NR | 200 | High | 1,00% (0.168 – 3.264) |
| Boudet-Berquier, 2017 | France | Europe | Cohort | NR | Adult | Pre-gestational BMI Underweight <18.5 kg/m2 (WHO, 2000) | Collected from medical records | 3368 | Low | 0,12% (0.039 – 0.300) |
| Chaurasia, 2021 | India | Asia | Cohort | First | Adult and adolescent | Pre-gestational BMI Underweight <18.5 kg/m2 (IOM, 2009) | NR | 100 | High | 1,00% (0.050 – 4.832) |
| Fortner, 2009 | USA | North America | Cohort | NR | Adult and adolescent | Pre-gestational BMI Underweight <19.8 kg/m2 (IOM, 1990) | Systolic blood pressure ≥ 140 mmHg or diastolic blood pressure ≥ 90 mmHg after 20 weeks of gestation | 1043 | Low | 0,10% (0.004 – 0.480) |
| Gaillard, 2011 | Netherlands | Europe | Cohort | All | Adult | Pre-gestational BMI Underweight <20 kg/m2 (NR Criteria) | Systolic blood pressure ≥ 140 mmHg or diastolic blood pressure ≥ 90 mmHg after 20 weeks of gestation | 6902 | High | 0,34% (0.227 – 0.509) |
| Kumari, 2024 | India | Asia | Cohort | All | Adult | Pre-gestational BMI Underweight <18.5 kg/m2 (IOM, 2009) | Blood pressure exceeded 140/90 mmHg on two occasions, at least 4 hours apart | 100 | High | 1% (0.05 – 4.832) |
| Lautredou, 2022 | France | Europe | Cohort | Second and third | Adult | Pre-gestational BMI Underweight <18.5 kg/m2 (IOM, 2009) | 2013 American College of Obstetricians and Gynecologists criteria | 3162 | High | 0,06% (0.010 – 0.208) |
| Ogunyemi, 1998 | USA | North America | Cohort | First | Adult and adolescent | Pre-gestational BMI Underweight <19.8 kg/m2 (IOM, 1990) | Reported by the pregnant woman | 582 | High | 0,17% (0.008 – 0.844) |
| Saito, 2021 | Japan | Asia | Cohort | All | Adult | Pre-gestational BMI Underweight <18.5 kg/m2 (Japanese Criteria) | Systolic blood pressure ≥ 140 mmHg or diastolic blood pressure ≥ 90 mmHg after 20 weeks of gestation | 85228 | High | 0,18% (0.056 – 0.431) |
| Shimko, 2019 | Slovakia | Europe | Cohort | All | Adult | Pre-gestational BMI Underweight <18.5 kg/m2 (IOM, 2009) | Systolic blood pressure ≥ 140 mmHg or diastolic blood pressure ≥ 90 mmHg after 20 weeks of gestation | 7122 | High | 0,08% (0.033 – 0.175) |
| Sugimura, 2020 | Japan | Asia | Cohort | All | Adult | Pre-gestational BMI Underweight <18.5 kg/m2 (IOM, 2009) | Systolic blood pressure ≥ 140 mmHg or diastolic blood pressure ≥ 90 mmHg after 20 weeks of gestation | 6066 | High | 0,48% (0.326 – 0.676) |
| Sun, 2020 | China | Asia | Cohort | All | Adult | Pre-gestational BMI Underweight <18.5 kg/m2 (WHO,2004) | NR | 3172 | High | 0,16% (0.057 – 0.349) |
| Vaca-Merino, 2022 | Ecuador | South America | Cross-sectional | All | Adult and adolescent | Pre-gestational BMI Underweight <20 kg/m2 (OMS) | NR | 894 | High | 0,22% (0.037 – 0.737) |
| Vince, 2020 | Croatia | Europe | Cross-sectional | All | Adult and adolescent | Pre-gestational BMI Underweight <18.5 kg/m2 (IOM, 2009) | Systolic blood pressure ≥ 140 mmHg or diastolic blood pressure ≥ 90 mmHg after 20 weeks of gestation | 32051 | Low | 0,03% (0.016 – 0.055) |
| Wang, 2019 | China | Asia | Cohort | Second and third | Adult | Pre-gestational BMI Underweight <18.5 kg/m2 (IOM, 2009) | Systolic blood pressure ≥ 140 mmHg or diastolic blood pressure ≥ 90 mmHg after 20 weeks of gestation | 8926 | Low | 0,49% (0.362 – 0.655) |

Legend: NR – Not Reported

**Table S3** - Summary of characteristics of included studies that presented data on the prevalence of overweight/obesity and anemia coexistence.

| **Author/Year** | **Country** | **Continent** | | **Study Design** | | **Gestational Trimester** | **Life Cicle** | **Sample Size** | **BMI** | **Anemia Diagnostic Criteria** | **Risk of Bias (High or Low)** | **Prevalence of overweight/obesity, and anemia (%)** |
| --- | --- | --- | --- | --- | --- | --- | --- | --- | --- | --- | --- | --- |
| Ahmed, 2025 | Rwanda | | Africa | Cross-sectional | Second and third | | Adult | 445 | Pre-gestational BMI Overweight/obesity ≥25 kg/m2 (WHO, 2016) | Hb < 11 g/dl (WHO) | Low | 15.28% (12.16 - 18.85) |
| Ali, 2025 | Bangladesh | | Asia | Cross-sectional | Third | | Adult | 150 | Pre-gestational BMI Overweight/obesity (Criteria NR) | NR | High | 8% (4.407 – 13.2) |
| Amarasinghe, 2022 | Sri Lanka | | Asia | Cross-sectional | First | | Adult and adolescent | 3127 | Pre-gestational BMIOverweight/obesity ≥25 kg/m2 (WHO, 2022) | Hb < 11 g/dl (WHO) | Low | 6,13% (5,316 – 7,025) |
| Andola, 2021 | India | | Asia | Cohort | Third | | Adult and adolescent | 200 | Pre-gestational BMI Overweight/obesity (Criteria NR) | NR | High | 11,50% (7.613 – 16.5) |
| Bhowmik, 2019 | Bangladesh | | Asia | Cohort | First | | Adult | 498 | Gestational BMI Overweight/obesity ≥ 23 kg/m2 (WHO, 2000) | Hb < 11 g/dl (WHO) | High | 3,61% (2.224 – 5.544) |
| Deshmukh, 2016 | India | | Asia | Cohort | First | | Adult | 500 | Pre-gestational BMI Overweight/obesity ≥25 kg/m2 (WHO, 2011) | NR | High | 1.80% (0.88 – 3.277) |
| El-Gilany, 2009 | Saudi Arabia | | Asia | Cohort | First | | Adult and adolescent | 787 | Pre-gestational BMI Overweight/obesity ≥25 kg/m2 (WHO, 1995) | Hb<10.5 | High | 22,62% (19.8 – 25.64) |
| Elmugabil, 2016 | Sudan | | Africa | Cross-sectional | First | | Adult | 388 | Pre-gestational BMI Overweight/obesity ≥25 kg/m2 (WHO) | Hb < 11 g/dl (WHO) | High | 30,18% (25.46 – 35.23) |
| Laddad, 2018 | India | | Asia | Cohort | First | | Adult | 1500 | Pre-gestational BMI Overweight/obesity ≥25 kg/m2 (WHO, 2015) | NR | High | 3.73% (2.86 – 4.786) |
| Mardones, 2008 | Chile | | South America | Cross-sectional | All | | Adult | 1782 | Pre-gestational BMI Overweight/obesity (WHO criteria) | Hb < 11 g/dl (WHO) | Low | 6,34% (5.279 – 7.546) |
| Oliveira, 2016 | Brazil | | South America | Cross-sectional | All | | Adult and adolescent | 129 | Gestational BMI Overweight/obesity (Atalah et al., 1997) | Hb < 11 g/dl (WHO) | High | 16,32% (10.65 – 23.41) |
| Park, 2010 | South Korea | | Asia | Cohort | NR | | NR | 2311 | Pre-gestational BMI Overweight/obesity ≥ 23 kg/m2 (WHO, 2000) | Hb < 11 g/dl (WHO) | High | 0,27% (0.068 – 0.734) |
| Razzak, 2021 | Pakistan | | Asia | Cohort | All | | Adult and adolescent | 1000 | Pre-gestational BMI Overweight/obesity ≥ 23 kg/m2 (WHO, 2000) | NR | High | 4,80% (3.6 – 6.262) |
| Sahu, 2007 | India | | Asia | Cohort | Second | | Adult | 380 | Pre-gestational BMI Overweight/obesity ≥25 kg/m2 (NR Criteria) | Hb < 11 g/dl (WHO) | Low | 2.11% (0.982 – 3.958) |
| Santos, 2020 | Brazil | | South America | Cross-sectional | Third | | Adult and adolescent | 461 | Gestational BMI Overweight/obesity (NR Criteria) | Hb < 11 g/dl (WHO) | High | 8,24% (5.984 – 11.03) |
| Sun, 2020 | China | | Asia | Cohort | All | | Adult | 3172 | Pre-gestational BMI Overweight/obesity (NR criteria) | Hb < 11 g/dl (WHO) | High | 2,24% (1.765 – 2.799) |
| Tan, 2018 | China | | Asia | Cohort | All | | Adult and adolescent | 11782 | Pre-gestational BMI Overweight/obesity ≥25 kg/m2 (WHO) | IDA Test Serum ferritin concentration <20 µg/L and Hemoglobin level <110 g/L | Low | 0,95% (0.786 – 1.138) |
| Ugwuja, 2010 | Nigeria | | Africa | Cross-sectional | Second | | Adult | 349 | Pre-gestational BMI Overweight/obesity ≥25 kg/m2 (Abrams et. al., 1995) | Hb < 11 g/dl (WHO) | High | 40.40% (35.34 – 45.62) |

Legend: NR – Not Reported

**Table S4 –** Summary of characteristics of included studies that presented data on the prevalence of overweight and vitamin D deficiency coexistence

| **Author/Year** | | **Country** | **Continent** | **Study Design** | **Gestational Trimester** | **Life Cicle** | **BMI** | **Vitamin D deficiency diagnostic criteria** | **Sample Size** | **Risk of Bias (High or Low)** | **Prevalence of overweight/obesity and vitamin D deficiency (%)** |
| --- | --- | --- | --- | --- | --- | --- | --- | --- | --- | --- | --- |
| Bhowmik, 2019 | Bangladesh | | Asia | Cohort | First | Adult | Gestational BMI Overweight/obesity ≥ 23 kg/m2 (WHO, 2000) | < 30 nmol/l | 498 | High | 8,84% (6.573 – 11.58) |
| Choi, 2015 | South Korea | | Asia | Cohort | All | Adult | Pre-gestational BMI Overweight/obesity ≥ 24 kg/m2 (Wildmar et al., 2004) | <20 ng/mL | 220 | High | 9.09% (5.801 – 13.45) |
| Daraki, 2018 | Greece | | Europe | Cohort | First | Adult and adolescent | Pre-gestational BMI Overweight/obesity ≥ 25 kg/m2 (OMS) | <37.7 nmol/L (~15 ng/mL) | 532 | Low | 14.47% (11.67 – 17.66) |
| Gómez, 2016 | Uruguay | | South America | Cross-sectional | Third | Adult and adolescent | Pre-gestational BMI Overweight/obesity ≥ 25 kg/m2 (NR Criteria) | Severe deficiency: Vitamin D concentration < 10 ng/ml | 71 | High | 33,80% (23.54 – 45.36) |
| Huang, 2023 | Taiwan | | Asia | Cross-sectional | All | Adult and adolescent | Pre-gestational BMI Overweight/obesity ≥ 25 kg/m2 (NR Criteria) | 25(OH)D less than 20 ng/mL | 1502 | Low | 5.06% (4.035 – 6.258) |
| Lee, 2017 | Malaysia | | Asia | Cohort | Third | Adult | Pre-gestational BMI Overweight/obesity ≥ 24 kg/m2 (NR Criteria) | <20 ng/mL | 575 | Low | 53.57% (49.48 – 57.62) |
| Lusawat, 2018 | India | | Asia | Cross-sectional | Second and Third | Adult and adolescent | Gestational BMI Overweight/obesity ≥ 23 kg/m2 (WHO, 2004) | <20 ng/mL | 75 | Low | 42.67% (31.85 – 54.04) |
| Morales, 2014 | Spain | | Europe | Cohort | First | Adult and adolescent | Pre-gestational BMI Overweight/obesity ≥ 25 kg/m2 (NR Criteria) | <20 ng/mL | 2489 | Low | 6.19% (5.274 – 7.222) |
| Rodriguez-Dehli, 2015 | Spain | | Europe | Cohort | First | Adult and adolescent | Pre-gestational BMI Overweight/obesity ≥ 25 kg/m2 (NR Criteria) | <20 ng/mL (50 nmol/L) | 453 | High | 10.60% (8.006 – 13.69) |
| Yadav, 2023 | India | | Asia | Cross-sectional | First and Second | Adult | Pre-gestational BMI Overweight/obesity ≥ 25 kg/m2 (NR Criteria) | <20 ng/mL | 160 | High | 35.00% (27.91 – 42.63) |

**Table S5 -** Summary of characteristics of included studies that presented data on the prevalence of overweight and vitamin B12 deficiency coexistence

| **Author/Year** | **Country** | **Continent** | **Study Design** | **Gestational Trimester** | **Life Cicle** | **BMI** | **Vitamin B12 deficiency diagnostic criteria** | **Sample Size** | **Risk of Bias (High or Low)** | **Prevalence of overweight/obesity and vitamin B12 deficiency (%)** |
| --- | --- | --- | --- | --- | --- | --- | --- | --- | --- | --- |
| Bhowmik, 2019 | Bangladesh | Asia | Cohort | First | Adult | Gestational BMI Overweight/obesity ≥ 23 kg/m2 (WHO, 2000) | Vitamin B12 deficiency (< 200 pg/ml) | 498 | High | 2,01% (1.024 – 3.55) |
| Sukumar, 2016 | United Kingdom | Europe | Retrospective Case-control | Third | Adult | Pre-gestational BMI Obesity ≥ 30 kg/m2 (NR Criteria) | <150 pmol/L | 344 | High | 12,79% (9.566 – 16.64) |

**Table S6 -** Summary of characteristics of included studies that presented data on prevalence of overweight/obesity and folate deficiency coexistence

| **Author/Year** | **Country** | **Continent** | **Study Design** | **Gestational Trimester** | **Life Cicle** | **BMI** | **Folate Deficiency diagnostic criteria** | **Sample Size** | **Risk of Bias (High or Low)** | **Prevalence of overweight/obesity and folate deficiency (%)** |
| --- | --- | --- | --- | --- | --- | --- | --- | --- | --- | --- |
| Bhowmik, 2019 | Bangladesh | Asia | Cohort | First | Adult | Gestational BMI Overweight/obesity ≥ 23 kg/m2 (WHO, 2000) | Folate deficiency (< 3 ng/ml) | 498 | High | 0,40% (0.067 – 1.321) |
| Sukumar, 2016 | United Kingdom | Europe | Retrospective Case-control | Third | Adult | Pre-gestational BMI Obesity ≥ 30 kg/m2 (NR Criteria) | <7 nmol/L | 344 | High | 1,16% (0.370 – 2.781) |
