# Supplementary material for: Double Burden of Malnutrition Prevalence in Pregnant Women: Systematic Review and Meta‐Analysis
Source: J Hum Nutr Diet. 2026 Sep 17;39(5):e70347. doi: 10.1111/jhn.70347 (PMC13586418; doi:10.1111/jhn.70347)
Supplement: Supplementary file 4 [file JHN-39-0-s006.docx]

*Appendix S5* **Risk of bias for each study assessed by Joanna Briggs Institute critical appraisal checklist.**

**Cohort Studies**

| **Author** | **Year of Publication** | **Criteria 1** | **Criteria 2** | **Criteria 3** | **Criteria 4** | **Criteria 5** | **Criteria 6** | **Criteria 7** | **Criteria 8** | **Criteria 9** | **Criteria 10** | **Criteria 11** | **Risk of Bias** | **Obs.** |
| --- | --- | --- | --- | --- | --- | --- | --- | --- | --- | --- | --- | --- | --- | --- |
| Andola | 2021 | Y | Y | Y | U | N | Y | Y | Y | U | N | Y | High | No clear strategy for confounders |
| Boudet-Berquier | 2017 | Y | Y | Y | Y | Y | Y | Y | Y | Y | Y | Y | Low |  |
| Chaurasia | 2021 | Y | Y | Y | Y | N | Y | Y | N | N | N | Y | High | No clear strategy for confounders; Follow-up incomplete |
| Chen | 2022 | Y | Y | Y | Y | Y | Y | Y | Y | Y | N | Y | High | Incomplete follow-up strategies |
| Ekin | 2017 | Y | Y | Y | Y | N | Y | Y | Y | Y | N | Y | High | Confounding strategies unclear; Follow-up incomplete |
| El-Gilany | 2009 | Y | Y | Y | Y | Y | Y | Y | N | N | N | Y | High | Follow-up duration insufficient |
| Fortner | 2009 | Y | Y | Y | Y | Y | Y | Y | Y | Y | Y | Y | Low |  |
| Gaillard | 2011 | Y | Y | Y | Y | Y | Y | Y | N | N | N | Y | High | Follow-up time insufficient |
| Hu | 2021 | Y | Y | Y | Y | Y | Y | Y | Y | Y | N | Y | High | Incomplete follow-up strategies |
| Hung | 2016 | Y | Y | Y | Y | N | Y | Y | Y | Y | N | Y | High | Confounding strategies unclear; Follow-up incomplete |
| Kim | 2021 | Y | Y | Y | Y | Y | Y | Y | Y | Y | Y | Y | Low |  |
| Lackovic | 2021 | Y | Y | Y | Y | Y | Y | Y | N | N | N | Y | High | Follow-up time insufficient |
| Lan | 2019 | Y | Y | Y | Y | N | Y | Y | N | N | N | Y | High | Confounding strategies unclear; Follow-up incomplete |
| Lautredou | 2022 | Y | Y | Y | Y | Y | Y | Y | Y | Y | N | Y | High | Incomplete follow-up strategies |
| Li | 2018 | Y | Y | Y | Y | Y | Y | Y | Y | Y | N | Y | High | Incomplete follow-up strategies |
| Lyu | 2024 | Y | Y | Y | Y | Y | Y | Y | Y | Y | Y | Y | Low |  |
| Ma | 2024 | Y | Y | Y | Y | Y | Y | Y | Y | Y | Y | Y | Low |  |
| Menon | 2016 | Y | Y | Y | Y | Y | Y | N | N | Y | Y | Y | High | Incomplete follow-up strategies |
| Mote | 2024 | Y | Y | Y | Y | Y | Y | Y | Y | N | N | Y | High | Incomplete follow-up strategies |
| Ogunyemi | 1998 | Y | Y | Y | Y | N | Y | Y | N | N | N | Y | High | Confounding strategies unclear; Follow-up insufficient |
| Park | 2010 | Y | Y | Y | Y | Y | Y | Y | Y | Y | N | Y | High | Incomplete follow-up strategies |
| Razzak | 2021 | Y | Y | Y | Y | Y | Y | Y | U | U | Y | Y | High | there is no mention of how the follow-up was and the strategies for incomplete follow-up |
| Saito | 2021 | Y | Y | Y | Y | Y | Y | Y | Y | Y | N | Y | High | Incomplete follow-up strategies |
| Shimko | 2019 | Y | Y | Y | Y | U | Y | Y | Y | N | N | Y | High | Confounding strategies unclear; Follow-up incomplete |
| Song | 2022 | Y | Y | Y | Y | Y | Y | Y | Y | N | N | Y | High | Follow-up time insufficient |
| Sugimura | 2020 | Y | Y | Y | Y | Y | Y | Y | N | N | N | Y | High | Follow-up time insufficient; No clear strategy for follow-up incomplete |
| Sun | 2022 | Y | Y | Y | Y | Y | Y | Y | Y | Y | N | Y | High | Incomplete follow-up strategies |
| Sun | 2020 | Y | Y | Y | Y | Y | Y | Y | Y | N | N | Y | High | Incomplete follow-up strategies |
| Tan | 2018 | Y | Y | Y | Y | Y | Y | Y | Y | Y | Y | Y | Low |  |
| Teshome | 2021 | Y | Y | Y | Y | Y | Y | Y | Y | Y | N | Y | High | Incomplete follow-up strategies |
| Tranidou | 2023 | Y | Y | Y | Y | N | Y | Y | Y | N | N | Y | High | No clear strategy for confounders; Follow-up incomplete |
| Wang | 2019 | Y | Y | Y | Y | Y | Y | Y | Y | Y | Y | Y | Low |  |
| Wu | 2022 | Y | Y | Y | Y | Y | Y | Y | Y | Y | Y | Y | Low |  |
| Yang | 2023 | Y | Y | Y | Y | Y | Y | Y | Y | Y | Y | Y | Low |  |
| Yong | 2022 | Y | Y | Y | Y | Y | Y | Y | Y | Y | Y | Y | Low |  |
| Bhowmik | 2019 | Y | Y | Y | Y | Y | Y | Y | Y | N | N | Y | High | Incomplete follow-up strategies |
| Kumari | 2024 | Y | Y | N | Y | Y | Y | Y | Y | U | N | Y | High | Incomplete follow-up strategies |
| Laddad | 2018 | N | Y | Y | Y | Y | Y | N | Y | Y | U | Y | High | Diagnostic criteria are unclear |
| Sahu | 2007 | Y | Y | Y | Y | Y | Y | Y | Y | Y | Y | Y | Low |  |
| Deshmukh | 2016 | N | Y | Y | Y | U | Y | Y | Y | Y | U | Y | High | Groups were not similar |
| Rodriguez-Dehli | 2015 | Y | y | Y | Y | Y | Y | Y | N | Y | N | Y | High | Non-similar groups; No clear strategy for follow-up incomplete |
| Choi | 2015 | Y | Y | Y | Y | Y | N | Y | Y | Y | N | Y | High | No clear strategy for follow-up incomplete |
| Lee | 2017 | Y | Y | Y | Y | Y | Y | Y | Y | Y | Y | Y | Low |  |
| Morales | 2014 | Y | Y | Y | Y | Y | Y | Y | Y | Y | Y | Y | Low |  |
| Daraki | 2018 | Y | Y | Y | Y | Y | Y | Y | Y | Y | Y | Y | Low |  |

| **Risk of bias for each individual study assessed by Joanna Briggs Institute critical appraisal checklist for cohort studies.** | | |
| --- | --- | --- |
| Y = Yes, N = No, U = Unclear, NA = Not applicable |  |  |
| 1.     Were the two groups similar and recruited from the same population? |  |  |
| 2.     Were the exposures measured similarly to assign people to both exposed and unexposed groups? |  |  |
| 3.     Was the exposure measured in a valid and reliable way? |  |  |
| 4.     Were confounding factors identified? |  |  |
| 5.     Were strategies to deal with confounding factors stated? |  |  |
| 6.     Were the groups/participants free of the outcome at the start of the study (or at the moment of exposure)? |  |  |
| 7.     Were the outcomes measured in a valid and reliable way? |  |  |
| 8.     Was the follow up time reported and sufficient to be long enough for outcomes to occur? |  |  |
| 9.     Was follow up complete, and if not, were the reasons to loss to follow up described and explored? |  |  |
| 10.  Were strategies to address incomplete follow up utilized? | | |
| 11.  Was appropriate statistical analysis used? | | |

**Cross-sectional Studies**

| **Author** | **Year of Publication** | **Criteria 1** | **Criteria 2** | **Criteria 3** | **Criteria 4** | **Criteria 5** | **Criteria 6** | **Criteria 7** | **Criteria 8** | **Risk of Bias** | **Obs.** |
| --- | --- | --- | --- | --- | --- | --- | --- | --- | --- | --- | --- |
| Abreu | 2019 | Y | Y | Y | Y | Y | Y | Y | Y | Low |  |
| Amarasinghe | 2022 | Y | Y | Y | Y | Y | Y | Y | Y | Low |  |
| Elmugabil | 2016 | Y | Y | Y | Y | N | N | Y | Y | High | Confounding factors were not fully identified or addressed. |
| Gómez | 2016 | Y | Y | Y | Y | N | N | Y | Y | High | No clear strategy to address confounding factors. |
| Khair | 2023 | Y | Y | Y | Y | N | N | Y | Y | High | Confounding factors not fully identified or addressed. |
| Mardones | 2008 | Y | Y | Y | Y | Y | Y | Y | Y | Low |  |
| Oliveira | 2016 | Y | Y | Y | Y | N | N | Y | Y | High | Confounding factors not fully identified or addressed. |
| Vaca-Merino | 2022 | Y | Y | Y | Y | N | N | Y | Y | High | Confounding factors not fully identified or addressed. |
| Vince | 2020 | Y | Y | Y | Y | Y | Y | Y | Y | Low |  |
| Santos | 2020 | Y | Y | Y | Y | N | N | Y | Y | High | Confounding factors not fully identified or addressed. |
| Ahmed | 2025 | Y | Y | Y | Y | Y | Y | Y | Y | Low |  |
| Ali | 2025 | U | N | U | N | N | N | Y | Y | High | Confounding factors not fully identified or addressed. |
| Deshmukh | 2016 | Y | Y | Y | Y | Y | N | Y | Y | High | Confounding factors not fully identified or addressed. |
| Yadav | 2023 | Y | Y | Y | Y | Y | N | Y | Y | High | Confounding factors not fully identified or addressed. |
| Lusawat | 2018 | Y | Y | Y | Y | Y | Y | Y | Y | Low |  |
| Huang | 2023 | Y | Y | Y | Y | Y | Y | Y | Y | Low |  |

| **Risk of bias for each individual study assessed by Joanna Briggs Institute critical appraisal checklist for cross-sectional studies.** | | |
| --- | --- | --- |
| Y = Yes, N = No, U = Unclear, NA = Not applicable |  |  |
| 1.     Were the criteria for inclusion in the sample clearly defined? |  |  |
| 2.     Were the study subjects and the setting described in detail? |  |  |
| 3.     Was the exposure measured in a valid and reliable way? |  |  |
| 4.     Were objective, standard criteria used for measurement of the condition? |  |  |
| 5.     Were confounding factors identified? |  |  |
| 6.     Were strategies to deal with confounding factors stated? |  |  |
| 7.     Were the outcomes measured in a valid and reliable way? |  |  |
| 8.     Was appropriate statistical analysis used? |  |  |

**Case-control studies**

| **Author** | **Year of Publication** | **Criteria 1** | **Criteria 2** | **Criteria 3** | **Criteria 4** | **Criteria 5** | **Criteria 6** | **Criteria 7** | **Criteria 8** | **Criteria 9** | **Criteria 10** | **Risk of Bias** | **Obs.** |
| --- | --- | --- | --- | --- | --- | --- | --- | --- | --- | --- | --- | --- | --- |
| Sukumar | 2016 | N | N | N | Y | Y | Y | Y | Y | Y | Y | High | Non-comparable groups |

**Risk of bias for each individual study assessed by Joanna Briggs Institute critical appraisal checklist for case-control studies.**

| Y = Yes, N = No, U = Unclear, NA = Not applicable |  |
| --- | --- |
| 1.     Were the groups comparable other than the presence of disease in cases or the absence of disease in controls? |  |
| 2.     Were cases and controls matched appropriately? |  |
| 3.     Were the same criteria used for identification of cases and controls? |  |
| 4.     Was exposure measured in a standard, valid and reliable way? |  |
| 5.     Was exposure measured in the same way for cases and controls? |  |
| 6.     Were confounding factors identified? |  |
| 7.     Were strategies to deal with confounding factors stated? |  |
| 8.     Were outcomes assessed in a standard, valid and reliable way for cases and controls? |  |
| 9.     Was the exposure period of interest long enough to be meaningful? |  |
| 10.  Was appropriate statistical analysis used? | |
