# Supplementary material for: Double Burden of Malnutrition Prevalence in Pregnant Women: Systematic Review and Meta‐Analysis
Source: J Hum Nutr Diet. 2026 Sep 17;39(5):e70347. doi: 10.1111/jhn.70347 (PMC13586418; doi:10.1111/jhn.70347)
Supplement: Supplementary file 5 [file JHN-39-0-s007.docx]

**Figure S6 -** Risk of bias in the included studies (The Joanna Briggs Institute Critical Appraisal checklist for cohort studies).

**Figure S7 -** Risk of bias in the included studies (The Joanna Briggs Institute Critical Appraisal checklist for cross-sectional studies).
