# Supplementary material for: Double Burden of Malnutrition Prevalence in Pregnant Women: Systematic Review and Meta‐Analysis
Source: J Hum Nutr Diet. 2026 Sep 17;39(5):e70347. doi: 10.1111/jhn.70347 (PMC13586418; doi:10.1111/jhn.70347)
Supplement: Supplementary file 6 [file JHN-39-0-s008.docx]

**Figure S2 -** Prevalence of underweight and GDM in pregnant women for subgroups.

**Figure S3 -** Prevalence of underweight and hypertension in pregnant women for subgroups.

**Figure S4 -** Prevalence of overweight and anemia in pregnant women for subgroups.

**Figure S5** - Prevalence of overweight/obesity and vitamin D deficiency in pregnant women for subgroups.
