# Supplementary material for: Double Burden of Malnutrition Prevalence in Pregnant Women: Systematic Review and Meta‐Analysis
Source: J Hum Nutr Diet. 2026 Sep 17;39(5):e70347. doi: 10.1111/jhn.70347 (PMC13586418; doi:10.1111/jhn.70347)
Supplement: Supplementary file 8 [file JHN-39-0-s004.doc]

Appendix S4 **Excluded articles and reasons for exclusion**

| **AUTHOR** | **PUBLICATION YEAR** | **REASON FOR EXCLUSION** |
| --- | --- | --- |
| Agrawal (1) | 2011 | (1) Use secondary data |
| Barišić (2) | 2017 | (1) Use secondary data |
| Bhattacharya (3) | 2007 | (1) Use secondary data |
| Bone (4) | 2023 | (1) Use secondary data |
| Chen (5) | 2020 | (1) Use secondary data |
| Cheng (6) | 2022 | (1) Use secondary data |
| Chen-Xu (7) | 2022 | (1) Use secondary data |
| Chung (8) | 2012 | (1) Use secondary data |
| Denison (9) | 2014 | (1) Use secondary data |
| Ehrenberg (10) | 2004 | (1) Use secondary data |
| El-Chaar (11) | 2011 | (1) Use secondary data |
| Frey (12) | 2022 | (1) Use secondary data |
| Gong (13) | 2023 | (1) Use secondary data |
| González-Plaza (14) | 2022 | (1) Use secondary data |
| Hauger (15) | 2008 | (1) Use secondary data |
| Jiang (16) | 2017 | (1) Use secondary data |
| Karasneh (17) | 2021 | (1) Use secondary data |
| Katon (18) | 2013 | (1) Use secondary data |
| Kim (19) | 2011 | (1) Use secondary data |
| Knight-Agarwal (20) | 2016 | (1) Use secondary data |
| Kusrini (21) | 2021 | (1) Use secondary data |
| Leung (22) | 2008 | (1) Use secondary data |
| Li (23) | 2013 | (1) Use secondary data |
| Liu (24) | 2014 | (1) Use secondary data |
| Liu (25) | 2009 | (1) Use secondary data |
| Locks (26) | 2020 | (1) Use secondary data |
| Madzia (27) | 2020 | (1) Use secondary data |
| Magann (28) | 2013 | (1) Use secondary data |
| Mayasari (29) | 2021 | (1) Use secondary data |
| Miao (30) | 2023 | (1) Use secondary data |
| Simas (31) | 2019 | (1) Use secondary data |
| Mrema (32) | 2018 | (1) Use secondary data |
| Muhuza (33) | 2023 | (1) Use secondary data |
| Nonterah (34) | 2019 | (1) Use secondary data |
| Perea (35) | 2022 | (1) Use secondary data |
| Qi (36) | 2020 | (1) Use secondary data |
| Rosinha (37) | 2021 | (1) Use secondary data |
| Scott-Pillai (38) | 2013 | (1) Use secondary data |
| Sebastián (39) | 2012 | (1) Use secondary data |
| Shi (40) | 2022 | (1) Use secondary data |
| Sorbye (41) | 2017 | (1) Use secondary data |
| Thrift (42) | 2014 | (1) Use secondary data |
| Trojner Bregar (43) | 2016 | (1) Use secondary data |
| Wang (44) | 2018 | (1) Use secondary data |
| Wang (45) | 2021 | (1) Use secondary data |
| Wei (46) | 2015 | (1) Use secondary data |
| Weschenfelder (47) | 2021 | (1) Use secondary data |
| Wirawan (48) | 2022 | (1) Use secondary data |
| Xu (49) | 2022 | (1) Use secondary data |
| Zhang (50) | 2022 | (1) Use secondary data |
| LIFECYCLE PROJECT (51) | 2019 | (1) Use secondary data |
| Kiljan (52) | 2025 | (1) Use secondary data |
| Zheng (53) | 2025 | (1) Use secondary data |
| May (54) | 2025 | (1) Use secondary data |
| Li (55) | 2025 | (1) Use secondary data |
| Marchewka-Długońska (56) | 2025 | (1) Use secondary data |
| Bone (57) | 2025 | (1) Use secondary data |
| Ciulei (58) | 2024 | (1) Use secondary data |
| Freitas-Costa (59) | 2025 | (1) Use secondary data |
| Iwama (60) | 2025 | (1) Use secondary data |
| Liu (61) | 2024 | (1) Use secondary data |
| Adikari (62) | 2016 | (2) Don't have the necessary information |
| Amoakoh-Coleman (63) | 2017 | (2) Don't have the necessary information |
| Ayensu (64) | 2020 | (2) Don't have the necessary information |
| Guo (65) | 2024 | (2) Don't have the necessary information |
| Hong (66) | 2022 | (2) Don't have the necessary information |
| Ito (67) | 2023 | (2) Don't have the necessary information |
| Kanwal (68) | 2022 | (2) Don't have the necessary information |
| Khan (69) | 2023 | (2) Don't have the necessary information |
| Krishnan (70) | 2020 | (2) Don't have the necessary information |
| Kumera (71) | 2018 | (2) Don't have the necessary information |
| Lannoo (72) | 2023 | (2) Don't have the necessary information |
| Liu (73) | 2019 | (2) Don't have the necessary information |
| Luccisano (74) | 2021 | (2) Don't have the necessary information |
| Masho (75) | 2015 | (2) Don't have the necessary information |
| Oleszczuk-Modzelewska (76) | 2022 | (2) Don't have the necessary information |
| Rezk (77) | 2015 | (2) Don't have the necessary information |
| Shaukat (78) | 2019 | (2) Don't have the necessary information |
| Singh (79) | 2023 | (2) Don't have the necessary information |
| Sámano (80) | 2021 | (2) Don't have the necessary information |
| Tsarna (81) | 2023 | (2) Don't have the necessary information |
| Abenhaim (82) | 2007 | (3) The author did not return with additional information or could not be found. |
| Adnan (83) | 2012 | ((3) The author did not return with additional information or could not be found. |
| Bouvier (84) | 2019 | (3) The author did not return with additional information or could not be found. |
| Chen (85) | 2021 | ((3) The author did not return with additional information or could not be found. |
| Choi (86) | 2021 | (3) The author did not return with additional information or could not be found. |
| Choi (87) | 2011 | (3) The author did not return with additional information or could not be found. |
| Feng (88) | 2021 | (3) The author did not return with additional information or could not be found. |
| Garmendia (89) | 2017 | (3) The author did not return with additional information or could not be found. |
| Liang (90) | 2019 | (3) The author did not return with additional information or could not be found. |
| Pedersen (91) | 2020 | (3) The author did not return with additional information or could not be found. |
| Rocha (92) | 2005 | (3) The author did not return with additional information or could not be found. |
| Rozário (93) | 2021 | (3) The author did not return with additional information or could not be found. |
| Sreelakshmy (94) | 2024 | (3) The author did not return with additional information or could not be found. |
| Tan (95) | 2020 | (3) The author did not return with additional information or could not be found. |
| Tebbani (96) | 2017 | (3) The author did not return with additional information or could not be found. |
| Vargas-Sanabria (97) | 2021 | (3) The author did not return with additional information or could not be found. |
| Kenea (98) | 2018 | (4) Pregnants with HIV |
| Ke (99) | 2023 | (5) Study with pregnant GDM only |
| Gaillard (100) | 2011 | (6) Duplicate |

REFERÊNCIAS

1.	Agrawal P, Mishra V, Agrawal S. Covariates of maternal overweight and obesity and the risk of adverse pregnancy outcomes: findings from a nationwide cross sectional survey. J Public Health. agosto de 2012;20(4):387–97.

2.	Barišić A, Finderle A. Pre-pregnancy body mass index and perinatal outcome. Med Flum [Internet]. 1^o^ de março de 2017 [citado 9 de novembro de 2025];53(1). Disponível em: http://hrcak.srce.hr/173374

3.	Bhattacharya S, Campbell DM, Liston WA, Bhattacharya S. Effect of Body Mass Index on pregnancy outcomes in nulliparous women delivering singleton babies. BMC Public Health. dezembro de 2007;7(1):168.

4.	Bone JN, Joseph KS, Magee LA, Muraca GM, Razaz N, Mayer C, et al. Prepregnancy body mass index and adverse perinatal outcomes in the presence of other maternal risk factors. AJOG Glob Rep. maio de 2023;3(2):100175.

5.	Chen CN, Chen HS, Hsu HC. Maternal Prepregnancy Body Mass Index, Gestational Weight Gain, and Risk of Adverse Perinatal Outcomes in Taiwan: A Population-Based Birth Cohort Study. Int J Environ Res Public Health. 14 de fevereiro de 2020;17(4):1221.

6.	Cheng. Factors associated with iron status and gestational anemia among pregnant women in northern Taiwan. 2022;

7.	Chen Xu J, Coelho Â. Association between Body Mass Index and Gestational Weight Gain with Obstetric and Neonatal Complications in Pregnant Women with Gestational Diabetes. Acta Médica Port. 26 de abril de 2022;35(10):718–28.

8.	Chung JH, Melsop KA, Gilbert WM, Caughey AB, Walker CK, Main EK. Increasing pre-pregnancy body mass index is predictive of a progressive escalation in adverse pregnancy outcomes. J Matern Fetal Neonatal Med. setembro de 2012;25(9):1635–9.

9.	Denison F, Norwood P, Bhattacharya S, Duffy A, Mahmood T, Morris C, et al. Association between maternal body mass index during pregnancy, short‐term morbidity, and increased health service costs: a population‐based study. BJOG Int J Obstet Gynaecol. janeiro de 2014;121(1):72–82.

10.	Ehrenberg HM, Durnwald CP, Catalano P, Mercer BM. The influence of obesity and diabetes on the risk of cesarean delivery. Am J Obstet Gynecol. setembro de 2004;191(3):969–74.

11.	El-Chaar D, Finkelstein SA, Tu X, Fell DB, Gaudet L, Sylvain J, et al. The Impact of Increasing Obesity Class on Obstetrical Outcomes. J Obstet Gynaecol Can. março de 2013;35(3):224–33.

12.	Frey HA, Ashmead R, Farmer A, Kim YH, Shellhaas C, Oza-Frank R, et al. Association of Prepregnancy Body Mass Index With Risk of Severe Maternal Morbidity and Mortality Among Medicaid Beneficiaries. JAMA Netw Open. 28 de junho de 2022;5(6):e2218986.

13.	Gong Y, Xu Y, Wan K, Wang Y, Zeng L, Zou K, et al. A prospective analysis of optimal total weight gain ranges and trimester-specific weight gain rates for Chinese pregnant women. BMC Pregnancy Childbirth. 24 de janeiro de 2023;23(1):60.

14.	González-Plaza E, Bellart J, Martínez-Verdú MÁ, Arranz Á, Luján-Barroso L, Seguranyes G. Pre-pregnancy overweight and obesity prevalence and relation to maternal and perinatal outcomes. Enferm Clínica Engl Ed. junho de 2022;32:S23–30.

15.	Hauger MS, Gibbons L, Vik T, Belizán JM. Prepregnancy weight status and the risk of adverse pregnancy outcome. Acta Obstet Gynecol Scand. setembro de 2008;87(9):953–9.

16.	Jiang S, Chipps D, Cheung WN, Mongelli M. Comparison of adverse pregnancy outcomes based on the new IADPSG 2010 gestational diabetes criteria and maternal body mass index. Aust N Z J Obstet Gynaecol. outubro de 2017;57(5):533–9.

17.	Karasneh RA, Migdady FH, Alzoubi KH, Al-Azzam SI, Khader YS, Nusair MB. Trends in maternal characteristics, and maternal and neonatal outcomes of women with gestational diabetes: A study from Jordan. Ann Med Surg [Internet]. julho de 2021 [citado 9 de novembro de 2025];67. Disponível em: https://journals.lww.com/10.1016/j.amsu.2021.102469

18.	Katon J, Reiber G, Williams MA, Yanez D, Miller E. Weight Loss After Diagnosis with Gestational Diabetes and Birth Weight Among Overweight and Obese Women. Matern Child Health J. fevereiro de 2013;17(2):374–83.

19.	Kim SY, England L, Wilson HG, Bish C, Satten GA, Dietz P. Percentage of Gestational Diabetes Mellitus Attributable to Overweight and Obesity.

20.	Knight-Agarwal CR, Williams LT, Davis D, Davey R, Cochrane T, Zhang H, et al. Association of BMI and interpregnancy BMI change with birth outcomes in an Australian obstetric population: a retrospective cohort study. BMJ Open. maio de 2016;6(5):e010667.

21.	Kusrini I, Kristanto Mulyantoro D, Tjandrarini DH, Ashar H. Profile of Double of Undernutrition Problem, Coexistence with Anemia among Pregnant Women Indonesia 2018: A Cross Sectional Survey. Open Access Maced J Med Sci [Internet]. 15 de novembro de 2021 [citado 9 de novembro de 2025];9(E). Disponível em: https://oamjms.eu/index.php/mjms/article/view/7052

22.	Leung T, Leung T, Sahota D, Chan O, Chan L, Fung T, et al. Trends in maternal obesity and associated risks of adverse pregnancy outcomes in a population of Chinese women. BJOG Int J Obstet Gynaecol. novembro de 2008;115(12):1529–37.

23.	Li N, Liu E, Guo J, Pan L, Li B, Wang P, et al. Maternal Prepregnancy Body Mass Index and Gestational Weight Gain on Pregnancy Outcomes. Jaddoe VWV, organizador. PLoS ONE. 20 de dezembro de 2013;8(12):e82310.

24.	Liu L, Hong Z, Zhang L. Associations of prepregnancy body mass index and gestational weight gain with pregnancy outcomes in nulliparous women delivering single live babies. Sci Rep. 5 de agosto de 2015;5(1):12863.

25.	Liu X, Du J, Wang G, Chen Z, Wang W, Xi Q. Effect of pre-pregnancy body mass index on adverse pregnancy outcome in north of China. Arch Gynecol Obstet. janeiro de 2011;283(1):65–70.

26.	Locks LM, Patel A, Katz E, Simmons E, Hibberd P. Seasonal trends and maternal characteristics as predictors of maternal undernutrition and low birthweight in Eastern Maharashtra, India. Matern Child Nutr. abril de 2021;17(2):e13087.

27.	Madzia J, McKinney D, Kelly E, DeFranco E. Influence of Gestational Weight Gain on the Risk of Preterm Birth for Underweight Women Living in Food Deserts. Am J Perinatol. agosto de 2021;38(S 01):e77–83.

28.	Magann EF, Doherty DA, Sandlin AT, Chauhan SP, Morrison JC. The effects of an increasing gradient of maternal obesity on pregnancy outcomes. Aust N Z J Obstet Gynaecol. junho de 2013;53(3):250–7.

29.	Mayasari NR, Hu TY, Chao JCJ, Bai CH, Chen YC, Huang YL, et al. Associations of the pre-pregnancy weight status with anaemia and the erythropoiesis-related micronutrient status. Public Health Nutr. dezembro de 2021;24(18):6247–57.

30.	Miao H, Liang F, Zheng Z, Chen H, Li X, Guo Y, et al. Weight progression and adherence to weight gain target in women with vs. without gestational diabetes: a retrospective cohort study. BMC Pregnancy Childbirth. 13 de julho de 2023;23(1):513.

31.	Moore Simas TA, Waring ME, Callaghan K, Leung K, Ward Harvey M, Buabbud A, et al. Weight gain in early pregnancy and risk of gestational diabetes mellitus among Latinas. Diabetes Metab. janeiro de 2019;45(1):26–31.

32.	Mrema D, Lie RT, Østbye T, Mahande MJ, Daltveit AK. The association between pre pregnancy body mass index and risk of preeclampsia: a registry based study from Tanzania. BMC Pregnancy Childbirth. dezembro de 2018;18(1):56.

33.	Muhuza MPU, Zhang L, Wu Q, Qi L, Chen D, Liang Z. The association between maternal HbA1c and adverse outcomes in gestational diabetes. Front Endocrinol. 16 de março de 2023;14:1105899.

34.	Nonterah EA, Adomolga E, Yidana A, Kagura J, Agorinya I, Ayamba EY, et al. Descriptive epidemiology of anaemia among pregnant women initiating antenatal care in rural Northern Ghana. Afr J Prim Health Care Fam Med [Internet]. 10 de abril de 2019 [citado 9 de novembro de 2025];11(1). Disponível em: http://www.phcfm.org/index.php/PHCFM/article/view/1892

35.	Perea V, Simó-Servat A, Quirós C, Alonso-Carril N, Valverde M, Urquizu X, et al. Role of Excessive Weight Gain During Gestation in the Risk of ADHD in Offspring of Women With Gestational Diabetes. J Clin Endocrinol Metab. 28 de setembro de 2022;107(10):e4203–11.

36.	Qi Y, Sun X, Tan J, Zhang G, Chen M, Xiong Y, et al. Excessive gestational weight gain in the first and second trimester is a risk factor for gestational diabetes mellitus among women pregnant with singletons: A repeated measures analysis. J Diabetes Investig. novembro de 2020;11(6):1651–60.

37.	Rosinha PMO, Dantas RAB, Alves MIP, Azevedo TCMF, Inácio IMR, Ferreira SGE, et al. Body mass index and gestational weight gain: relevance in gestational diabetes and outcomes - A retrospective cohort study. Arch Endocrinol Metab [Internet]. 19 de abril de 2022 [citado 9 de novembro de 2025]; Disponível em: https://www.aem-sbem.com/article/body-mass-index-and-gestational-weight-gain-relevance-in-gestational-diabetes-and-outcomes-a-retrospective-cohort-study/

38.	Scott‐Pillai R, Spence D, Cardwell C, Hunter A, Holmes V. The impact of body mass index on maternal and neonatal outcomes: a retrospective study in a UK obstetric population, 2004–2011. BJOG Int J Obstet Gynaecol. julho de 2013;120(8):932–9.

39.	Sebastián Manzanares G, Ángel Santalla H, Irene Vico Z, López Criado MS, Alicia Pineda L, José Luis Gallo V. Abnormal maternal body mass index and obstetric and neonatal outcome. J Matern Fetal Neonatal Med. março de 2012;25(3):308–12.

40.	Shi H, Chen L, Wang Y, Sun M, Guo Y, Ma S, et al. Severity of Anemia During Pregnancy and Adverse Maternal and Fetal Outcomes. JAMA Netw Open. 3 de fevereiro de 2022;5(2):e2147046.

41.	Sorbye LM, Skjaerven R, Klungsoyr K, Morken NH. Gestational diabetes mellitus and interpregnancy weight change: A population-based cohort study. Ma RCW, organizador. PLOS Med. 1^o^ de agosto de 2017;14(8):e1002367.

42.	Thrift AP, Callaway LK. The effect of obesity on pregnancy outcomes among Australian Indigenous and non‐Indigenous women. Med J Aust. novembro de 2014;201(10):592–5.

43.	Trojner Bregar A, Blickstein I, Bržan Šimenc G, Janša V, Verdenik I, Lučovnik M, et al. Perinatal Advantages and Disadvantages of Being Underweight before Pregnancy: A Population-Based Study. Gynecol Obstet Invest. 2017;82(3):303–6.

44.	Wang C, Lin L, Su R, Zhu W, Wei Y, Yan J, et al. Hemoglobin levels during the first trimester of pregnancy are associated with the risk of gestational diabetes mellitus, pre-eclampsia and preterm birth in Chinese women: a retrospective study. BMC Pregnancy Childbirth. dezembro de 2018;18(1):263.

45.	Wang T, Li L, Wu C, Cao R, Li Q, Yu L, et al. Body Mass Index and Gestational Weight Gain Are Associated with Maternal and Neonatal Outcomes Based on Chinese Women. Chiefari E, organizador. J Diabetes Res. 24 de novembro de 2021;2021:1–9.

46.	Wei YM, Yang HX, Zhu WW, Liu XY, Meng WY, Wang YQ, et al. Risk of adverse pregnancy outcomes stratified for pre-pregnancy body mass index. J Matern Fetal Neonatal Med. 2 de julho de 2016;29(13):2205–9.

47.	Weschenfelder F, Hein F, Lehmann T, Schleußner E, Groten T. Contributing Factors to Perinatal Outcome in Pregnancies with Gestational Diabetes—What Matters Most? A Retrospective Analysis. J Clin Med. 18 de janeiro de 2021;10(2):348.

48.	Wirawan F, Nurrika D. Maternal pre-pregnancy anemia and childhood anemia in Indonesia: a risk assessment using a population-based prospective longitudinal study. Epidemiol Health. 1^o^ de novembro de 2022;44:e2022100.

49.	Xu H, Hutcheon JA, Liu X, Stephansson O, Cnattingius S, Arkema EV, et al. Risk of gestational diabetes mellitus in relation to early pregnancy and gestational weight gain before diagnosis: A population‐based cohort study. Acta Obstet Gynecol Scand. novembro de 2022;101(11):1253–61.

50.	Zhang J, Wensheng A, Li L. The Association of Prepregnancy Body Mass Index with Pregnancy Outcomes in Chinese Women. J Diabetes Res. 2022;

51.	LifeCycle Project-Maternal Obesity and Childhood Outcomes Study Group, Voerman E, Santos S, Inskip H, Amiano P, Barros H, et al. Association of Gestational Weight Gain With Adverse Maternal and Infant Outcomes. JAMA. 7 de maio de 2019;321(17):1702.

52.	Kiljan M, Szablewska AW. Prenatal Physical Activity, Pre-Pregnancy BMI, and Their Relationship with Gestational Diabetes: A Retrospective-Prospective Single-Center Study. Nutrients. 24 de fevereiro de 2025;17(5):786.

53.	Zheng L, Liao Q, Liu J, Shi J, Chen Z, Lin L. Association between Pre-pregnancy BMI and Pregnancy Outcomes in Women with Gestational Diabetes: A Retrospective Cohort Study. Pak J Med Sci. 23 de dezembro de 2024;41(1):37–43.

54.	May L, Mason D, Van De Vyver M, Conradie M, Hall DR. Hypertensive disorders in a gestational diabetes cohort from Cape Town, South Africa. Pregnancy Hypertens. março de 2025;39:101185.

55.	Li H, Yin B, Jiang N, Zhu B. Effect of Combined Gestational Diabetes Mellitus and Preeclampsia on Pregnancy Outcomes. Clin Exp Obstet Gynecol. 24 de fevereiro de 2025;52(2):27065.

56.	Marchewka-Długońska J, Nieczuja-Dwojacka J, Krygowska K, Bogdanovich V, Sys D, Baranowska B, et al. Effects of pre-pregnancy BMI and gestational weight gain on pregnancy and neonatal outcomes in Poland. Sci Rep. 4 de março de 2025;15(1):7603.

57.	Bone JN, Joseph KS, Magee LA, Mayer C, Lisonkova S. Relationship between prepregnancy body mass index and gestational age–specific risk of stillbirth and perinatal death in women with chronic hypertension. Am J Obstet Gynecol. julho de 2025;233(1):61.e1-61.e15.

58.	Ciulei MA, Gallagher K, Ba DM, Beck C, Pobee RA, Gernand AD, et al. Iron status and anemia in a representative sample of US pregnant women is not associated with pre-pregnancy BMI: Results from the NHANES (1999–2010) study. Cardoso MA, organizador. PLOS ONE. 10 de setembro de 2024;19(9):e0300912.

59.	Freitas-Costa NCD, Carrilho TRB, Normando Da Costa P, Constante HM, Fujimori E, Sayuri Sato AP, et al. Hemoglobin Concentrations and Prevalence of Anemia During Pregnancy: Results from the Brazilian Maternal and Child Nutrition Consortium. Curr Dev Nutr. junho de 2025;9(6):107458.

60.	Iwama N, Yokoyama M, Yamashita H, Miyakoshi K, Yasuhi I, Kawasaki M, et al. Impact of maternal overweight/obesity and high fasting plasma glucose on adverse perinatal outcomes in early gestational diabetes mellitus. J Diabetes Investig. abril de 2025;16(4):744–54.

61.	Liu Y, Feng X, Wang X. Gestational weight gain trajectories in patients with gestational diabetes mellitus: a retrospective cohort study. Br J Hosp Med. 30 de agosto de 2024;85(8):1–14.

62.	Adikari AMNT, Sivakanesan R, Wijesinghe DGNG, Liyanage C. Assessment of nutritional status of pregnant women in a rural area in Sri Lanka. Trop Agric Res. 4 de julho de 2016;27(2):203.

63.	Amoakoh-Coleman M, Ogum-Alangea D, Modey-Amoah E, Ntumy MY, Adanu RM, Oppong SA. Blood pressure patterns and body mass index status in pregnancy: An assessment among women reporting for antenatal care at the Korle-Bu Teaching hospital, Ghana. Vinci MC, organizador. PLOS ONE. 6 de dezembro de 2017;12(12):e0188671.

64.	Ayensu J, Annan R, Lutterodt H, Edusei A, Peng LS. Prevalence of anaemia and low intake of dietary nutrients in pregnant women living in rural and urban areas in the Ashanti region of Ghana. Glover-Amengor M, organizador. PLOS ONE. 24 de janeiro de 2020;15(1):e0226026.

65.	Guo Y, Lu J, Bahani M, Ding G, Wang L, Zhang Y, et al. Triglyceride-glucose index in early pregnancy predicts the risk of gestational diabetes: a prospective cohort study. Lipids Health Dis. 25 de março de 2024;23(1):87.

66.	Hong M, Liang F, Zheng Z, Chen H, Guo Y, Li K, et al. Weight gain rate in the second and third trimesters and fetal growth in women with gestational diabetes mellitus: a retrospective cohort study. BMC Pregnancy Childbirth. dezembro de 2022;22(1):424.

67.	Ito M, Kyozuka H, Yamaguchi T, Sugeno M, Murata T, Hiraiwa T, et al. Association between Gestational Weight Gain and Risk of Hypertensive Disorders of Pregnancy among Women with Obesity: A Multicenter Retrospective Cohort Study in Japan. Nutrients. 23 de maio de 2023;15(11):2428.

68.	Kanwal A, Bashir A. Vitamin B12 in Pregnancy and its Relationship with Maternal BMI and Gestational Diabetes Mellitus. Pak J Med Sci [Internet]. 5 de março de 2022 [citado 9 de novembro de 2025];38(4). Disponível em: https://pjms.org.pk/index.php/pjms/article/view/5120

69.	Ali Khan I, Alhaizan MA, Neyazi SM, Al-Hakeem MM, Alshammary AF. Relevance of Serum Levels and Functional Genetic Variants in Vitamin D Receptor Gene among Saudi Women with Gestational Diabetes Mellitus. Nutrients. 8 de outubro de 2023;15(19):4288.

70.	Krishnan M, Murphy R, Okesene-Gafa KAM, Ji M, Thompson JMD, Taylor RS, et al. The Pacific-specific CREBRF rs373863828 allele protects against gestational diabetes mellitus in Māori and Pacific women with obesity. Diabetologia. outubro de 2020;63(10):2169–76.

71.	Kumera G, Gedle D, Alebel A, Feyera F, Eshetie S. Undernutrition and its association with socio-demographic, anemia and intestinal parasitic infection among pregnant women attending antenatal care at the University of Gondar Hospital, Northwest Ethiopia. Matern Health Neonatol Perinatol. dezembro de 2018;4(1):18.

72.	Lannoo L, Van Camp J, Brison N, Parijs I, Vancoillie L, Van Den Bogaert K, et al. What helps define outcomes in persistent uninterpretable non‐invasive prenatal testing: Maternal factors, fetal fraction or quality scores? Prenat Diagn. setembro de 2023;43(10):1333–43.

73.	Liu J, Song G, Meng T, Zhao G, Guo S. Weight retention at six weeks postpartum and the risk of gestational diabetes mellitus in a second pregnancy. BMC Pregnancy Childbirth. dezembro de 2019;19(1):272.

74.	Luccisano SP, Weber HC, Murfet GO, Robertson IK, Prior SJ, Hills AP. An Audit of Pre-Pregnancy Maternal Obesity and Diabetes Screening in Rural Regional Tasmania and Its Impact on Pregnancy and Neonatal Outcomes. Int J Environ Res Public Health. 16 de novembro de 2021;18(22):12006.

75.	Masho SW, Urban P, Cha S, Ramus R. Body Mass Index, Weight Gain, and Hypertensive Disorders in Pregnancy. Am J Hypertens. 1^o^ de junho de 2016;29(6):763–71.

76.	Oleszczuk-Modzelewska L, Malinowska-Polubiec A, Romejko-Wolniewicz E, Zawiejska A, Czajkowski K. What is the “cost” of reducing adverse pregnancy outcomes in patients with gestational diabetes mellitus - risk factors for perinatal complications in a retrospective cohort of pregnant women with GDM. BMC Pregnancy Childbirth. 19 de agosto de 2022;22(1):654.

77.	Rezk M, Marawan H, Dawood R, Masood A, Abo-Elnasr M. Prevalence and risk factors of iron-deficiency anaemia among pregnant women in rural districts of Menoufia governorate, Egypt. J Obstet Gynaecol. 3 de outubro de 2015;35(7):663–6.

78.	Shaukat S, Nur U. Effect of prepregnancy maternal BMI on adverse pregnancy and neonatal outcomes: results from a retrospective cohort study of a multiethnic population in Qatar. BMJ Open. setembro de 2019;9(9):e029757.

79.	Gupta M, Singh P, Kapoor N, Jindal S. Effect of High Prepregnancy Body Mass Index on Pregnancy Outcomes. J South Asian Fed Obstet Gynaecol. 31 de janeiro de 2023;14(6):685–9.

80.	Sámano R, Chico-Barba G, Flores-Quijano ME, Godínez-Martínez E, Martínez-Rojano H, Ortiz-Hernandez L, et al. Association of Pregestational BMI and Gestational Weight Gain with Maternal and Neonatal Outcomes in Adolescents and Adults from Mexico City. Int J Environ Res Public Health. 28 de dezembro de 2021;19(1):280.

81.	Tsarna E, Eleftheriades A, Tsomi E, Ziogou G, Vakas P, Panoskaltsis T, et al. The Role of Diet during Pregnancy in Protecting against Gestational Diabetes Mellitus in a Population with Mediterranean Dietary Habits: A Cross-Sectional Study. J Clin Med. 26 de fevereiro de 2023;12(5):1857.

82.	Abenhaim HA, Kinch RA, Morin L, Benjamin A, Usher R. Effect of prepregnancy body mass index categories on obstetrical and neonatal outcomes. Arch Gynecol Obstet. 21 de novembro de 2006;275(1):39–43.

83.	Adnan. The effects of maternal body mass index (BMI) on the pregnancy outcome among primigravida who delivered at Hospital Tengku Ampuan Afzan (HTAA), Kuantan, Pahang. 2012;

84.	Bouvier D, Forest JC, Dion-Buteau E, Bernard N, Bujold E, Pereira B, et al. Association of Maternal Weight and Gestational Weight Gain with Maternal and Neonate Outcomes: A Prospective Cohort Study. J Clin Med. 27 de novembro de 2019;8(12):2074.

85.	Chen YT, Zhang T, Chen C, Xia YY, Han TL, Chen XY, et al. Associations of early pregnancy BMI with adverse pregnancy outcomes and infant neurocognitive development. Sci Rep. 15 de fevereiro de 2021;11(1):3793.

86.	Choi H, Lim JY, Lim NK, Ryu HM, Kwak DW, Chung JH, et al. Impact of pre-pregnancy body mass index and gestational weight gain on the risk of maternal and infant pregnancy complications in Korean women. Int J Obes. janeiro de 2022;46(1):59–67.

87.	Choi SK, Park IY, Shin J chul. The effects of pre-pregnancy body mass index and gestational weight gain on perinatal outcomes in Korean women: a retrospective cohort study. Reprod Biol Endocrinol. dezembro de 2011;9(1):6.

88.	Feng. Effect of pre-pregnancy body mass index and gestational weight gain on perinatal outcomes. 2021;

89.	Garmendia ML, Zamudio C, Araya M, Kain J. Association between prepregnancy obesity and metabolic risk in Chilean premenopausal women 10 y postpartum. Nutrition. junho de 2017;38:20–7.

90.	Liang. Associations of Pre-pregnancy Body Mass Index and Gestational Weight Gain with Gestational Diabetes Mellitus: a Cohort Study in Southwest China. 2019;

91.	Pedersen DC, Bjerregaard LG, Nohr EA, Rasmussen KM, Baker JL. Associations of childhood BMI and change in BMI from childhood to adulthood with risks of hypertensive disorders in pregnancy. Am J Clin Nutr. novembro de 2020;112(5):1180–7.

92.	Rocha DDS, Netto MP, Priore SE, Lima NMMD, Rosado LEFPDL, Franceschini SDCC. Estado nutricional e anemia ferropriva em gestantes: relação com o peso da criança ao nascer. Rev Nutr. agosto de 2005;18(4):481–9.

93.	Rozário. The additive effect of interpregnancy interval and maternal body mass index on pregnancy induced hypertension in the U.S. 2021;

94.	Sreelakshmy. Association of pre-pregnancy body mass index to gestational weight gain, diabetes mellitus and hypertension. 2024;

95.	Tan ML, Chin YS, Lim PY, Ahmad Fuzi SF. Associations of obstetrical characteristics and dietary intakes with iron status among pregnant women in Selangor and Kuala Lumpur. Br Food J. 21 de julho de 2020;122(10):3115–27.

96.	Tebbani F, Oulamara H, Agli A. Effects of gestational weight gain on pregnancy complications. Nutr Clin Métabolisme. fevereiro de 2018;32(1):27–32.

97.	Vargas-Sanabria AP, Niño Tovar MA, Acosta Rodriguez AL, Acosta Rodriguez AL, Perez LA. Relación entre obesidad gestacional y desenlaces perinatales adversos: Estudio multicéntrico. Andes Pediatr. 23 de agosto de 2021;92(4):548–55.

98.	Kenea A, Negash E, Bacha L, Wakgari N. Magnitude of Anemia and Associated Factors among Pregnant Women Attending Antenatal Care in Public Hospitals of Ilu Abba Bora Zone, South West Ethiopia: A Cross-Sectional Study. Anemia. 12 de novembro de 2018;2018:1–7.

99.	Ke JF, Liu S, Ge RL, Ma L, Li MF. Associations of maternal pre-pregnancy BMI and gestational weight gain with the risks of adverse pregnancy outcomes in Chinese women with gestational diabetes mellitus. BMC Pregnancy Childbirth. 3 de junho de 2023;23(1):414.

100.	Gaillard R, Bakker R, Willemsen SP, Hofman A, Steegers EAP, Jaddoe VWV. Blood pressure tracking during pregnancy and the risk of gestational hypertensive disorders: The Generation R Study. Eur Heart J. dezembro de 2011;32(24):3088–97.
